# The geometry and genetics of hybridization

**DOI:** 10.1111/evo.14116

**Published:** 2020-11-23

**Authors:** Hilde Schneemann, Bianca De Sanctis, Denis Roze, Nicolas Bierne, John J. Welch

**Affiliations:** ^1^ Department of Genetics University of Cambridge Downing Street Cambridge United Kingdom; ^2^ Institut des Sciences de l'Évolution Université Montpellier UMR 5554, Montpellier Cedex 05 France; ^3^ Department of Zoology University of Cambridge Downing Street Cambridge United Kingdom; ^4^ CNRS, UMI 3614 Evolutionary Biology and Ecology of Algae Roscoff France; ^5^ Station Biologique de Roscoff Sorbonne Université Roscoff 29688 France; ^6^ ISEM Université Montpellier, CNRS, EPHE, IRD Montpellier France

**Keywords:** Fisher's geometric model, hybrid fitness, line crosses, quantitative genetics, speciation

## Abstract

When divergent populations form hybrids, hybrid fitness can vary with genome composition, current environmental conditions, and the divergence history of the populations. We develop analytical predictions for hybrid fitness, which incorporate all three factors. The predictions are based on Fisher's geometric model, and apply to a wide range of population genetic parameter regimes and divergence conditions, including allopatry and parapatry, local adaptation, and drift. Results show that hybrid fitness can be decomposed into intrinsic effects of admixture and heterozygosity, and extrinsic effects of the (local) adaptedness of the parental lines. Effect sizes are determined by a handful of geometric distances, which have a simple biological interpretation. These distances also reflect the mode and amount of divergence, such that there is convergence toward a characteristic pattern of intrinsic isolation. We next connect our results to the quantitative genetics of line crosses in variable or patchy environments. This means that the geometrical distances can be estimated from cross data, and provides a simple interpretation of the “composite effects.” Finally, we develop extensions to the model, involving selectively induced disequilibria, and variable phenotypic dominance. The geometry of fitness landscapes provides a unifying framework for understanding speciation, and wider patterns of hybrid fitness.

When genetically distinct populations meet and mate, their divergent alleles are brought together in new combinations. The fitness of these novel genotypes will influence the outcome of the hybridization, which might be as various as population fusion, hybrid speciation, or reinforcement selection for new prezygotic barriers.

Broadly speaking, the fitness of hybrids might depend on three factors. The first factor is the type of hybrid genotype. For example, the initial F1 cross is often fitter than subsequent crosses, and even than the parental lines (Frankel 1983; Price and Bouvier 2002; Escobar et al. 2008; Fraïsse et al. 2016). The second factor is the environmental conditions in which the hybrid is formed (Bordenstein and Drapeau 2001). For example, when parental lines are adapted to different habitats, hybrids might be selected against in those habitats, and yet enjoy a selective advantage in novel environments, due to transgressive variation (Moore 1977; Yakimowski and Rieseberg 2014). The third factor is the divergence history of the parental lines. This includes not only the amount of divergence (Bateson 1978; Waser 1993; Edmands 2002), but also how it was accrued. For example, beneficial heterosis is often observed when parental lines have been subject to severe inbreeding, such that the divergence comprises partially recessive deleterious mutations (Wright 1922; Neal 1935). This third factor implies that the outcome of hybridization might be used to make inferences about the divergence history (Dobzhansky 1937; Lynch 1991; Rundle and Whitlock 2001; Gavrilets 2004; Welch 2004; Demuth and Wade 2005; Rosas et al. 2010; Fraïsse et al. 2016; Yamaguchi and Otto 2020).

To study all of these factors, and the interactions between them, one approach uses the quantitative genetics of line crosses (Cockerham 1980; Hill 1982; Lynch 1991; Lynch and Walsh 1998, chs. 9‐10; Rundle and Whitlock 2001; Demuth and Wade 2005). This approach is fully general, and widely applied, but it does have limitations. With the quantitative genetics of single populations, a large body of theory can help us to interpret the variance components (Hill et al. 2008; Mäki‐Tanila and Hill 2014; Barton 2017; Walsh and Lynch 2018), but with line crosses, the “composite effects” are more difficult to interpret. For this reason, a lot of research on hybridization uses fitness landscapes (Dobzhansky 1937; Hill 1982; Orr 1995; Gavrilets 2004). With this alternative approach, clear understanding can come from simple models, with a few, biologically meaningful parameters. But such models can be difficult to fit to data, and often apply to a limited range of cases (such as the evolution of intrinsic incompatibilities).

Here, following previous authors, we combine these two approaches (Lynch 1991; Demuth and Wade 2005; Yamaguchi and Otto 2020), drawing an explicit connection between the quantitative genetics of line crosses (Hill 1982; Rundle and Whitlock 2001), and a class of fitness landscapes based on Fisher's geometric model (Fisher 1930, ch. 2). Fisher's model is a well‐studied model of both divergence (Hartl and Taubes 1998; Orr 1998b; Walsh and Lynch 2018, ch. 27) and hybridization, and it can account for a large number of empirical patterns (Mani and Clarke 1990; Barton 2001; Rosas et al. 2010; Chevin et al. 2014; Fraïsse et al. 2016; Simon et al. 2018; Thompson et al. 2019; Yamaguchi and Otto 2020). As such, it allows us to explore the effects of hybrid genome composition, environmental heterogeneity, and parental divergence history, all in a common framework.

This article is in three parts. In Part 1, we rederive analytical predictions from Fisher's model (Simon et al. 2018), and show that they apply to very wide range of divergence conditions and population genetic parameter regimes. We also present the results in a new way, to clarify the geometrical and biological meaning of the key quantities. In Part 2, we connect Fisher's model to quantitative genetics, providing a simple interpretation of the composite effects. We then express results for standard line crosses in different environments, unifying results from previous studies (Wright 1922; Lynch 1991; Hatfield and Schluter 1999; Rundle and Whitlock 2001; Chevin et al. 2014; Simon et al. 2018; Yamaguchi and Otto 2020). Finally, in Part 3, we introduce two extensions to the model, involving selectively induced associations between heterospecific alleles, and phenotypic dominance. These extensions address cases where the simplest model gives misleading or implausible predictions. We end by discussing some implications of our results for understanding the process of speciation.

## Fisher's Geometric Model and Hybridization

### MODEL DESCRIPTION AND NOTATION

#### Description of hybrid genotypes

We consider hybrids between two diploid parental lines P1 and P2, which differ by d substitutions. For simplicity, we ignore genetic variation within the parental lines at the time of hybridization, so that d is equal to the genetic distance between hybridizing individuals. This will be a reasonable approximation if within‐line variation is much smaller than between‐line divergence.

Hybrids will contain some combination of alleles from the two parental lines. We characterize hybrid genotypes in terms of their heterozygosity, $p_{12}$, and hybrid index, h. The heterozygosity is the proportion of the d divergent sites where the hybrid carries one allele from each line; the hybrid index is the total proportion of the divergent alleles that come from line P2. As such, h ranges from 0 for a pure P1 genotype, to 1 for a pure P2 genotype. We will also use the notation $p_{1}$ and $p_{2}$ to refer to the proportion of divergent sites that are homozygous for alleles from P1 and P2, such that $p_{1}+p_{2}+p_{12}=1$, and

(1) $$h\equiv p_{2}+\frac{1}{2}p_{12}$$

(Turelli and Orr 2000; Simon et al. 2018). Our overall aim is to show how hybrid fitness varies with h and $p_{12}$, and how this variation might be determined by environmental conditions and the history of parental divergence.

#### Fisher's model as a fitness landscape

Under Fisher's geometric model, each genotype is associated with the values of n continuously varying phenotypic traits, and so it can be represented as a point in an n‐dimensional trait space: $z=(z_{1},z_{2},\ldots ,z_{n})$. The fitness of the genotype depends on the distance of its phenotype from an optimal phenotype: $o=(o_{1},o_{2},\ldots ,o_{n})$. We use a weighted Euclidean distance:

(2) $$\begin{array}{cc} {\left\|z-o\right\|}_{\lambda } & \equiv \sqrt{\sum_{i=1}^{n}\lambda_{i}{\left(z_{i}-o_{i}\right)}^{2}}, \end{array}$$

where the $\lambda_{i}$ determine the strength of selection on each trait. Fitness is a decreasing function of distance (Turelli and Moyle 2007; Simon et al. 2018), such as

(3) $$\begin{array}{cc} \ln\;w & =-\alpha {\left\|z-o\right\|}_{\lambda }^{k}, \end{array}$$

where α is a constant, and *k* denotes the curvature of the fitness landscape, that is, how quickly fitness declines with the distance from the optimum (Peck et al. 1997; Tenaillon et al. 2007; Fraïsse et al. 2016; Fraïsse and Welch 2019). This model assumes a single phenotypic optimum at any given time and location, but the position of the optimum can vary in space and time, so that we can investigate divergence and hybridization under different environmental conditions.

How seriously should we view this simple phenotypic model? Although one or a few of the traits might be identified with real‐world quantitative traits, which might be measured in the field (Barton 1990; Thompson 2019), it is usually a mistake to treat all *n* of the traits in this way. Instead, Fisher's model is best viewed as an approximation to phenotypic models that are more realistic but less tractable (such as models of gene‐regulatory networks, as studied in systems biology; Martin 2014; Schiffman and Ralph 2017; Fraïsse and Welch 2019), or simply as a mapping between genotype and fitness. Under this interpretation, *n* is a parameter of the distribution of fitness effects, with no explicit phenotypic interpretation (and no effect on our major results—see below). Nevertheless, as we show below, thinking about the trait values is a useful way of deriving and interpreting testable predictions about hybrid fitness.

#### Hybrids under Fisher's model

**Figure 1:**
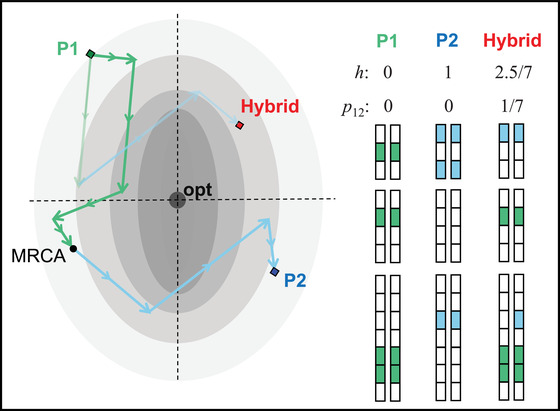
Under Fisher's geometric model, each genotype is associated with the values of n quantitative traits (illustrated with n=2), and its fitness depends on the distance of this phenotype from an optimum. The position of the optimum can change over time and space. Shown are two parental lines, P1 and P2, which differ by d=7 substitutions, each represented by a vector, denoted $m_{j}$ for j=1..d. These vectors represent the effects of the P2 allele, whether this is derived or ancestral. But they are ordered so that the chain passes through the most recent common ancestor (MRCA) of P1 and P2. Also shown are cartoons of the parental genomes, with the derived alleles as colored boxes. Hybrid genomes contain a mix of the parental alleles. In the hybrid shown, 1/7 of the divergent sites contains an allele from each line, so that $p_{12}=1/7$; and two further P2 alleles are present as homozygotes (one ancestral and one derived), yielding a hybrid index of h=2.5/7. The illustration shows that fixed differences can be physically linked. Such linkage reduces the variance in the hybrid indexes within a given cross, but plays no other role in our analyses.

Figure 1 shows how Fisher's model can be used to study hybridization. Under the model, the parental lines, P1 and P2, are represented by points in n‐dimensional trait space. Each of the d substitutions, which differentiate the lines, are represented as n‐dimensional vectors of change, notated $m_{j}$ for j=1…d. In any given genome, each of these substitutions can appear in either homozygous or heterozygous form. These substitutions accrued during the evolutionary divergence of P1 and P2 from their most recent common ancestor (MRCA), and so they can be represented as a chain, passing through the ancestral phenotype (Fig. 1). However, there is no important distinction between ancestral and derived alleles in this model, and so we define the direction of each substitution such that the chain starts at P1.

With this definition, the P2 genotype contains all of the substitutions in homozygous state, and so its phenotype on trait i=1…n can be written as

(4) $$z_{P2,i}=z_{P1,i}+\sum_{j=1}^{d}m_{\mathrm{ij}}$$

where $z_{P1,i}$ denotes the value of trait i in parental line P1, and the $m_{ij}$ describe the effects on trait i of introducing substitution j (i.e., the P2 allele, whether derived or ancestral). The phenotype of any given genotype can be written in the same way:

(5) $$z_{i}=z_{P1,i}+\sum_{j\in J_{\text{hom}}}m_{\mathrm{ij}}+\sum_{j\in J_{\text{het}}}\frac{1}{2}m_{\mathrm{ij}}$$

where $J_{\text{hom}}$ denotes the subset of $d\times p_{2}$ loci that are homozygous for the P2 allele, and $J_{\text{het}}$ denotes the nonoverlapping subset of $d\times p_{12}$ loci that are heterozygous.

The key assumption of equations (4) and (5) is that all substitutions act additively on all traits, both within and between loci. This means that the phenotypic effects of a substitution will not depend on the genomic background (although its fitness effects can vary). To make equation (5) useful, we will make a second key assumption: that $J_{\text{hom}}$ and $J_{\text{het}}$ can be treated as randomly chosen subsets of loci. Both of these assumptions—phenotypic additivity, and random choice of loci in hybrids—play a major role in the results below, but both are relaxed in the final section.

#### A useful distance measure

Results below will concern fitness, but in most cases, we will not work with fitness directly (Turelli and Orr 2000; Demuth and Wade 2005; Turelli and Moyle 2007). Instead, following Simon et al. (2018), we express most results in terms of a scaled squared distance to the optimum, which for brevity we will call “distance”. In particular, for a hybrid H, with phenotype $z_{H}$ (as defined via eq. 5), this distance is defined as

(6) $$r_{\mathrm{HO}}^{2}\equiv 4\frac{{\left\|z_{H}-o\right\|}_{\lambda }^{2}}{d{\left\|1\right\|}_{\lambda }^{2}}$$

where 1 is the unit vector, so that the scaling factor is

(7) $${\left\|1\right\|}_{\lambda }^{2}=\sum_{i=1}^{n}\lambda_{i}$$

**Figure 2:**
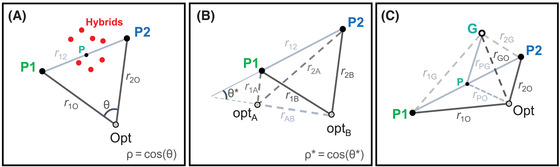
Predictions for hybrid fitness depend on a small number of geometric quantities. The distances are defined in some multi‐dimensional trait space, but are estimable, in principle. (A) With additive phenotypes, and a single environmental optimum, predictions depend on just three distances: the distances of the two parental phenotypes from the optimum ($r_{1O}^{2}$ and $r_{2O}^{2}$), and the distance between the parental phenotypes ($r_{12}^{2}$). Results can also be written in terms of ρ, which measures the extent to which the parental populations are maladapted to the current environment in similar ways. Also shown is the midparental phenotype, P. This is the expected phenotype of balanced hybrids, and so hybrid advantage is maximized when P coincides with the optimum. (B) With two environments, A and B, characterized by different optima, one measure of local adaptation is $\rho^{*}$: the cosine similarity between the vectors linking the optima, and the parental phenotypes. When the two parental phenotypes are close to the two optima, results depend on $r_{12}^{2}$ alone. (C) With variable phenotypic dominance, results depend on the phenotype of the global heterozygote, G, which is equivalent to the initial F1 cross under strictly biparental inheritance and expression, and which may differ from the midparental phenotype, P. In the example shown, G is closer to the P2 phenotype than the P1 phenotype, this implies directional dominance, with P2 alleles being dominant on average.

The major purpose of this scaling is to remove any dependence on parameters such as n and the $\lambda_{i}$, so that our results depend solely on distances. The most important distances are between the optimum and the two parental phenotypes; these are illustrated in Figure 2A.

(8) $$\begin{array}{ccc} r_{1O}^{2}\equiv 4\frac{{\left\|z_{P1}-o\right\|}_{\lambda }^{2}}{d{\left\|1\right\|}_{\lambda }^{2}} & & r_{2O}^{2}\equiv 4\frac{{\left\|z_{P2}-o\right\|}_{\lambda }^{2}}{d{\left\|1\right\|}_{\lambda }^{2}} \end{array}$$

Each of these distances is a scaled transformed fitness (eq. 3), or, equivalently, a measure of maladaptation to the current environmental conditions, such that larger distances correspond to lower fitness. We return below to the interpretation of the scaling factor.

### THE BROWNIAN BRIDGE APPROXIMATION

Simon et al. (2018) presented an approximation for the expected distance from the optimum of a hybrid with a given hybrid index and heterozygosity. This quantity is notated as $E(r_{\mathrm{HO}}^{2}|h,p_{12})$, where the expectation is defined over all hybrid genotypes with the same values of h and $p_{12}$. In this section, and Appendix 1 in the Supporting Information, we rederive this Brownian bridge approximation, and clarify its assumptions. We also present the key results in a new form, which clarifies their biological meaning (see next section).

The first assumption of the approximation is that the fixed effects on each trait, the $m_{ij}$, can be treated as realizations of normally distributed random variables with unit variances and no correlations between traits. This is justified by a transformation called simultaneous diagonalization, introduced in a very similar context by Martin and Lenormand (2006), and described in detail in Appendix 1 section A1.1 in the Supporting Information. The transformation assumes that the original distribution of effects is multivariate Gaussian (Waxman and Welch 2005; Martin and Lenormand 2006). However, if the divergence, d, is sufficiently large, then the summations in equation (5) lead to central‐limit‐type behavior, and so to approximate normality in a wider range of cases (Barton et al. 2017, section 3.2). After the transformation is applied, the $\lambda_{i}$ capture between‐trait differences in both the strength of selection, and the typical sizes of fixed factors.

Given statistical independence of the trait values, we have

(9) $$\begin{array}{cc} E\left(\left(r_{\mathrm{HO}}^{2}\right|h,p_{12}\right) & =4\frac{\sum \lambda_{i}\mathrm{Var}\left(z_{i}\right)}{d\sum \lambda_{i}}+4\frac{\sum \lambda_{i}E^{2}\left(z_{i}-o_{i}\right)}{d\sum \lambda_{i}} \end{array}$$

(10) ≡V+M

To derive the quantities V and M, we will next treat the $m_{ij}$ on each trait as the increments of a Brownian bridge, that is, a random walk or Brownian motion, constrained at each end by the parental phenotypes, and split into d equal steps (Revuz and Yor 1999; Simon et al. 2018). For the term V, which captures the variation in hybrid phenotypes, Appendix 1 section A1.2 in the Supporting Information shows that

(11) $$\begin{array}{cc} V & =4p_{2}\left(1-p_{2}\right)+p_{12}\left(1-p_{12}\right)-4p_{2}p_{12} \end{array}$$

(12) $$\begin{array}{c} =4h\left(1-h\right)-p_{12} \end{array}$$

where the three terms in equation (11), come from variation in the effects of homozygous P2 alleles, variation in the effects of heterozygous alleles, and a negative covariance term (because a given allele cannot appear in both homozygous and heterozygous state in the same genome).

For the term M, we note that the expected hybrid phenotype will lie on the line connecting the parental phenotypes (Figure 2A). Appendix 1 section A1.2 in the Supporting Information shows that

(13) $$\begin{array}{cc} M & \equiv {\left(1-h\right)}^{2}r_{1O}^{2}+h^{2}r_{2O}^{2}+2h\left(1-h\right)r_{1O}r_{2O}\rho \end{array}$$

(14) $$=r_{.O}^{2}+\left(h-\frac{1}{2}\right)\left(r_{2O}^{2}-r_{1O}^{2}\right)-h\left(1-h\right)r_{12}^{2}$$

where we have used equation (8), and the notation $r_{.O}^{2}\equiv \frac{1}{2}\left(r_{1O}^{2}+r_{2O}^{2}\right)$. We also use two new quantities that are illustrated in Figure 2A. The first quantity, ρ, which appears in equation (13), is the “cosine similarity” of the vectors connecting the parental phenotypes to the optimum. ρ can vary between 1, when these vectors point in the same direction, and −1, when they point in opposite directions (see also eq. 41 in the Supporting Information). The second quantity, $r_{12}^{2}$, which appears in equation (14), is the distance between the parental phenotypes:

(15) $$r_{12}^{2}\equiv 4\frac{{\left\|z_{P1}-z_{P2}\right\|}_{\lambda }^{2}}{d{\left\|1\right\|}_{\lambda }^{2}}$$

ρ and $r_{12}^{2}$ are related to each other via the cosine rule

(16) $$r_{12}^{2}=r_{1O}^{2}+r_{2O}^{2}-2r_{1O}r_{2O}\rho \;\mathrm{where}-1\leq \rho \leq 1$$

We now have an expectation for the expected fitness of any type of hybrid, using only its genomic composition ($p_{12}$ and h), and the three distances, $r_{1O}^{2}$, $r_{2O}^{2}$, and $r_{12}^{2}$. Notably, we have assumed almost nothing about the history of divergence between the parental lines, nor their common ancestral state. One way to understand this is to imagine all the possible paths between P1 and P2 that could be obtained by shuffling the order of the d substitution steps. One of these paths is the true history of divergence, and passes through the MRCA, but hybrids can lie on any of the paths. As a result, the Brownian bridge approximation is based on the notion of a random walk, but it does not require that the true process of divergence resembled a random walk.

To verify that the Brownian Bridge approximation works well for a wide range of divergence histories, Appendix 2 in the Supporting Information presents an individual‐based simulation study. We simulated the divergence between diploid populations under a full population genetic model, varying the parameter regime, and the patterns of demographic and environmental change. Our simulations included divergence in allopatry, and in parapatry, with ongoing gene flow (Endler 1977). We also simulated different levels of drift, selection, standing variation, and recombination. Results show that the Brownian bridge approximation is robust in all cases.

### BIOLOGICAL INTERPRETATION

Let us now consider a group of hybrids that might vary in their values of h and $p_{12}$. Combining results above, the expected distance of these hybrids from the optimum is

(17) $$\begin{array}{cc} E\left(r_{\mathrm{HO}}^{2}\right)= & \;r_{.O}^{2}\\ & +\left(\bar{h}-\frac{1}{2}\right)\left(r_{2O}^{2}-r_{1O}^{2}\right)\\ & -{\bar{p}}_{12}\\ & +\left(\bar{h}\left(1-\bar{h}\right)-\mathrm{Var}\left(h\right)\right)\left(4-r_{12}^{2}\right) \end{array}$$

where ${\bar{p}}_{12}$ is the mean level of heterozygosity in the hybrids of interest, and $\bar{h}$ and Var(h) are the mean and variance of their hybrid indexes. All four of the terms in equation (17) have a clear biological interpretation. First, and simplest, $r_{.O}^{2}$ is the mean distance to the optimum of the parental lines, as measured in the environment where the hybrids were scored; it tells us that hybrids will be fitter, on average, if their parents are fitter, on average, in the current environment. The second term depends on the difference in the parental distances in the current environment: $\left(\bar{h}-\frac{1}{2}\right)\left(r_{2O}^{2}-r_{1O}^{2}\right)$; it tells us that hybrids will be fitter if they contain more alleles from the fitter parent. The third term, $-{\bar{p}}_{12}$, is an intrinsic benefit of heterozygosity; it states that, for any given value of h, hybrids are fitter when they are more heterozygous. This is a form of heterosis (Frankel 1983). The effect is “intrinsic” because, unlike the two previous terms, it does not depend on the current position of the environmental optimum.

The final term in equation (17) is the intrinsic effect of admixture. The level of admixture is measured by $\bar{h}\left(1-\bar{h}\right)-\mathrm{Var}\left(h\right)$. This means that admixture is low when most alleles come from one of the parental lines (such that $\bar{h}$ is close to 0 or 1), or when there is a mix of alleles in the population, but most individual genomes are close to one or other parental type, such that Var(h) is close to its maximal value: $\bar{h}\left(1-\bar{h}\right)$. The admixture level is highest for a collection of balanced hybrids, where h=1/2 for all individuals. The effect of admixture is “intrinsic,” because it depends on the distance $r_{12}^{2}$, which depends on the parental phenotypes, but not on the current position of the optimum (Fig. 2A; eq. 15). The effect changes qualitatively with the size of $r_{12}^{2}$. When $r_{12}^{2}<4$, admixture brings a net fitness cost. This reflects the breaking up of co‐adapted gene complexes in the parental lines (Lynch 1991; Wallace 1991; Simon et al. 2018). When $r_{12}^{2}>4$, admixture brings a net fitness benefit. This reflects the potential benefits of transgressive variation in hybrids (Yakimowski and Rieseberg 2014), and this benefit increases with $r_{12}^{2}$.

Although each of the four terms of equation (17) has a different interpretation, variation among them is constrained. This is because the distances $r_{1O}^{2}$, $r_{2O}^{2}$, and $r_{12}^{2}$ are connected to each other by the geometry (Fig. 2A; eq. 16). Most importantly, parental lines cannot be, at once, highly divergent phenotypically (such that $r_{12}^{2}\gg 1$), and close to the same environmental optimum (such that $r_{.O}^{2}\approx 0$). This implies that large benefits of admixture are impossible without large costs due to parental maladaptation. However, the relative sizes of these terms can vary, according to the current position of the optimum. For a given value of $r_{12}^{2}$, $E(r_{\mathrm{HO}}^{2})$ is minimized when the optimum matches the midparental phenotype, denoted P in Figure 2A. (In this case, $r_{1O}^{2}=r_{2O}^{2}$ and ρ=−1, such that, from eq. 16, $r_{.O}^{2}=\frac{1}{4}r_{12}^{2}$). This restates a result of Yamaguchi and Otto (2020), and also follows intuitively: hybrids will be fittest when the optimal phenotype is exactly intermediate between the parental phenotypes (Moore 1977).

### HYBRID FITNESS AND THE PROCESS OF DIVERGENCE

The results above show that the outcomes of hybridization depend strongly on the value of $r_{12}^{2}$. So what exactly does this quantity measure? The numerator of equation (15) is $∥z_{P1}-z_{P2}{∥}_{\lambda }^{2}$: the amount of phenotypic divergence that resulted from the genomic divergence between the parental lines. In Appendix 1 section A1.5 in the Supporting Information, we show that the denominator, ${d∥1∥}_{\lambda }^{2}$, is equivalent to the expected phenotypic divergence under an unconstrained random walk in phenotypic space, conditional on the walk having d steps, and a similar distribution of effect sizes to the observed data (as parameterized by the $\lambda_{i}$). For this reason, $r_{12}^{2}$ can be thought of as the observed amount of phenotypic divergence, divided by the expected amount of phenotypic evolution under a random walk.

**Figure 3:**
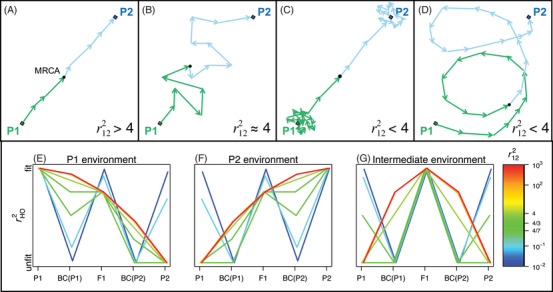
(A)–(D) the distance $r_{12}^{2}$ can vary systematically with the mode of divergence between the parents. The variation depends on the chain of d substitutions that differentiate the parental lines, and compares their trajectory to a random walk with the same number of steps, and distribution of effect sizes. (A) When substitutions form a more‐or‐less direct path between the parental phenotypes, the observed phenotypic difference is greater than would be predicted under a comparable random walk; this implies that $r_{12}^{2}>4$ with a maximum at $\max(r_{12}^{2})=4d$. (B) When the true path of divergence really did resemble a random walk, then $r_{12}^{2}\approx 4$ is expected. This might happen if stabilizing selection on the phenotype was ineffective, or if the optimum value wandered erratically. Systematically smaller values of $r_{12}^{2}$ are predicted under two conditions. Either (C) genomic divergence continued, despite effective stabilizing selection on the phenotype, leading to “system drift.” Or (D) populations successfully tracked environmental optima, but without leading to a straight path of substitutions. (E)–(G) $r_{12}^{2}$ also plays a key role in determining patterns of hybrid fitness, especially with locally adapted parents. Results are shown for the standard crosses, in three different environments, where the optimal phenotype coincides with: (E) the P1 phenotype, (F) the P2 phenotype, and (G) the midparental phenotype. Color shows variation in $r_{12}^{2}$, including the inflection points at $r_{12}^{2}=4/3$ (equal fitnesses for the F1 and fitter backcross), and $r_{12}^{2}=4/7$ (equal fitnesses for the less fit parent and the less fit backcross). All results use equation (17), with Var(h)=0, and the appropriate values of $\bar{h}$ and ${\bar{p}}_{12}$ for each cross; but results are shown on an arbitrary scale, such that fitter hybrids are higher on the plots.

As shown in Figure 3A–D, this implies that the value of $r_{12}^{2}$ contains some information about the mode of divergence between the populations.

For example, if $r_{12}^{2}>4$, then the parents show more phenotypic divergence than expected under a random walk. This implies that the d substitutions, which connect the parental phenotypes, form a chain with relatively little meandering or changing of direction. In terms of fitness effects, this implies that the P2 alleles are largely exchangeable, with all having similar effects on fitness in any given background. Indeed, we show in Appendix 1 section A1.5 in the Supporting Information that $r_{12}^{2}$ approaches a maximum of $\max(r_{12}^{2})=4d$, when the P2 alleles are completely exchangeable, causing changes of the same size and direction. This sort of pattern is unlikely to arise without positive selection, and so an observation of $r_{12}^{2}\gg 4$ suggests that the parental lines diverged via positive selection, either acting in one population alone, or in both populations, but in opposite directions in phenotypic space. This is illustrated in Figure 3A.

Similarly, an observation of $r_{12}^{2}\approx 4$ is expected if the parental phenotypes really did diverge by random‐walk‐like evolution (Fig. 3B). One way this might occur is if parental lines fixed mutations regardless of their fitness costs, for example, under severe inbreeding. It is notable that the intrinsic effects of admixture vanish when $r_{12}^{2}\approx 4$ (eq. 17). This agrees with the empirical observation that heterozygosity, rather than admixture level, is the major determinant of fitness in crosses between inbred lines (Wright 1922; Neal 1935; Simon et al. 2018).

Finally, if $r_{12}^{2}<4$, then populations have accrued less phenotypic divergence than would be expected under a random walk. This could occur in two quite distinct ways. First, stabilizing selection might maintain the phenotype at a (more‐or‐less) stationary optimum, while still allowing for divergence at the genomic level, perhaps by nearly neutral evolution (Barton 1989; Hartl and Taubes 1998). This process is closely related to “system drift” (Rosas et al. 2010; Schiffman and Ralph 2017), and is illustrated in Figure 3C. Alternatively, divergence could involve adaptation to a moving optimum, but without leading to a straight path of substitutions connecting P1 and P2. In the simplest case, this could arise if the two populations adapted, independently, to identical environmental change (Mani and Clarke 1990), because the chain of substitutions would then change direction as it passed through the common ancestor. A more complex example, involving an oscillating optimum, is illustrated in Figure 3D. In both cases, the result is a chain of substitutions whose start and end points are closer together than would be expected under a random walk, such that $r_{12}^{2}<4$.

Importantly, over large periods of time, at least one of these two processes—system drift, or environmental change that does not lead to a straight path—is very likely to occur. It is unlikely that populations will diverge for large periods by fixing exchangeable alleles. As such, at very large divergences, it becomes increasingly likely that $r_{12}^{2}\approx 0$ will hold. In fact, it follows directly from equations (8) and (15) that all of the key distances shown in Figure 2A will tend to vanish at large divergences, and we have the limit:

(18) $$\begin{array}{cc} \lim_{d\to \infty }E\left(r_{\mathrm{HO}}^{2}\right) & =4\bar{h}\left(1-\bar{h}\right)-4\mathrm{Var}\left(h\right)-{\bar{p}}_{12} \end{array}$$

where the scaling of equation (6) ensures that equation (18) is bounded at 0 and 1. Biologically, equation (18) implies that, as populations diverge genetically, both extrinsic fitness effects (as determined by $r_{1O}^{2}$ and $r_{2O}^{2}$), and any intrinsic benefits of admixture (as determined by $r_{12}^{2}$), will tend to become less and less important. The model predicts convergence to a characteristic pattern of intrinsic isolation between the parental lines, where a fixed cost of admixture is mitigated by a fixed benefit of heterozygosity.

In Appendix 2 in the Supporting Information, we present simulations supporting all of the arguments in this section, namely the relationship between $r_{12}^{2}$ and the mode of divergence, and the convergence over time to equation (18).

## FISHER'S MODEL AND THE QUANTITATIVE GENETICS OF LINE CROSSES

In this second part of the article, we show how the distances in phenotypic space, which govern the fitness of hybrids, relate to measurable quantities. To do this, we consider fitness measures from controlled crosses, which differ in their values of $\bar{h}$ and ${\bar{p}}_{12}$. These crosses include the initial F1 (P1×P2: $\bar{h}=1/2$, ${\bar{p}}_{12}=1$), the F2 (F1×F1: $\bar{h}={\bar{p}}_{12}=1/2$), and the reciprocal backcrosses (BC(P1)=F1×P1: $\bar{h}=1/4$, ${\bar{p}}_{12}=1/2$; and BC(P2)=F1×P2: $\bar{h}=3/4$, ${\bar{p}}_{12}=1/2$). The variance in the hybrid index, Var(h), depends on both the cross type, and the level of segregation and recombination (Lynch and Walsh 1998, ch. 9). In particular, if $\bar{c}$ is the mean rate of recombination among pairs of loci, then $\mathrm{Var}\left(h\right)\approx \left(1-2\bar{c}\right)/4$ among F1 gametes (Zeng et al. 1990; Lynch and Walsh 1998); and so, with random union of gametes, Var(h) will be half of this value for the F2, and a quarter of this value for backcrosses. However, $\bar{c}\approx 0.5$ for many species (Lynch and Walsh 1998, ch. 9), and in those cases, Var(h) can often be neglected.

### THE COMPOSITE EFFECTS UNDER FISHER'S MODEL

Let us begin by following Hill (1982; see also Lynch 1991; Lynch and Walsh 1998), and writing the expected value of an arbitrary trait in a cross as

(19) $$\begin{array}{cc} \mu \;= & \mu_{0}+\theta_{S}\left\{\alpha_{1}\right\}\;+\theta_{S}^{2}\left\{\alpha_{2}\right\}\;+\theta_{H}\left\{\delta_{1}\right\}\;+\theta_{H}^{2}\left\{\delta_{2}\right\}+\theta_{S}\theta_{H}\left\{\alpha_{1}\delta_{1}\right\}+\cdots \end{array}$$

where $\theta_{S}\equiv 1-2\bar{h}$ and $\theta_{H}\equiv 2{\bar{p}}_{12}-1$. The curly brackets contain the “composite effects,” which are defined in Table 1 (and noting that, in this standard notation, $\left\{\alpha_{1}\delta_{1}\right\}$ describes an interaction term, and not a product). Equation (19) shows only pairwise effects, but the model also includes higher order terms.

**Table 1:** Composite effects under Fisher's geometric model

	Composite effect	Geometric model prediction			
		Single environment	Two environments	Local adaptation	Phenotypic dominance
$\left\{\alpha_{1}\right\}$	additive	$\frac{1}{2}\left(r_{1O}^{2}-r_{2O}^{2}\right)$	$\frac{1}{2}\left(r_{1.}^{2}-r_{2.}^{2}\right)$	0	$\frac{1}{2}\left(r_{1O}^{2}-r_{2O}^{2}\right)-\frac{1}{4}\left(r_{1G}^{2}-r_{2G}^{2}\right)$
$\left\{\delta_{1}\right\}$	dominance	$-\frac{1}{2}$	$-\frac{1}{2}$	$-\frac{1}{2}$	$-\frac{1}{2}\left(1+r_{\mathrm{PO}}^{2}-r_{\mathrm{GO}}^{2}\right)$
$\left\{\alpha_{2}\right\}$	additive‐by‐additive epistasis	$\frac{1}{4}r_{12}^{2}-1$	$\frac{1}{4}r_{12}^{2}-1$	$\frac{1}{4}r_{12}^{2}-1$	$\frac{1}{4}r_{12}^{2}-1$
$\left\{\delta_{2}\right\}$	dominance‐by‐dominance	0	0	0	$\frac{1}{4}r_{\mathrm{GP}}^{2}-v$
$\left\{\alpha_{1}\delta_{1}\right\}$	additive‐by‐dominance	0	0	0	$-\frac{1}{4}\left(r_{1G}^{2}-r_{2G}^{2}\right)$
{ε}	environmental	–	$\frac{1}{2}\left(r_{.B}^{2}-r_{.A}^{2}\right)$	0	–
$\left\{\alpha_{1}\varepsilon \right\}$	additive‐by‐environmental	–	$\frac{1}{4}\left[\left(r_{1B}^{2}-r_{1A}^{2}\right)-\left(r_{2B}^{2}-r_{2A}^{2}\right)\right]$	$\frac{1}{2}r_{12}^{2}$	–
$\left\{\delta_{1}\varepsilon \right\}$	dominance‐by‐environmental	–	0	0	–
$\left\{\mu_{0}\right\}$	(intercept)	$\frac{1}{2}+r_{.O}^{2}-\frac{1}{4}r_{12}^{2}$	$\frac{1}{2}+r_{..}^{2}-\frac{1}{4}r_{12}^{2}$	$\frac{1}{2}+\frac{1}{4}r_{12}^{2}$	$\frac{1}{2}+v+r_{.O}^{2}-\frac{1}{4}r_{12}^{2}-\frac{1}{4}r_{\mathrm{GP}}^{2}+\frac{1}{2}\left(r_{\mathrm{GO}}^{2}-r_{\mathrm{PO}}^{2}\right)$

If we neglect Var(h), then both equations (17) and (19) are polynomials in $\bar{h}$ and ${\bar{p}}_{12}$. We can therefore choose “distance from the optimum” as the trait in equation (19). If we set $\mu =E(r_{\mathrm{HO}}^{2})$, and solve for the composite effects, the results are found in Table 1 (column “single environment”). Table 1 shows that Fisher's model predicts three nonzero composite effects. Their values reflect the biological distinctions discussed above. In particular, the additive effect, $\left\{\alpha_{1}\right\}=\frac{1}{2}\left(r_{1O}^{2}-r_{2O}^{2}\right)$, captures the benefits of carrying alleles from the fitter parent, whereas the dominance effect, $\left\{\delta_{1}\right\}=-\frac{1}{2}$, captures the intrinsic benefits of heterozygosity. The pairwise epistatic effect, $\left\{\alpha_{2}\right\}=\frac{1}{4}r_{12}^{2}-1$, balances the intrinsic costs and benefits of admixture.

Rundle and Whitlock (2001) presented a useful extension of equation (19) for traits scored in two environments. Introducing an indicator variable, I, which is 0 for individuals scored in “environment A,” and 1 for individuals scored in “environment B,” and defining $\theta_{E}\equiv 2I-1$, their model contains the additional terms:

(20) $$\begin{array}{cc} \mu_{\mathrm{AB}}= & \mu +\theta_{E}\left\{\varepsilon \right\}+\theta_{E}\theta_{S}\left\{\alpha_{1}\varepsilon \right\}+\theta_{E}\theta_{H}\left\{\delta_{1}\varepsilon \right\}+\cdots \end{array}$$

Fisher's geometric model is trivially extended in the same way, by adding a second environment, with a distinct optimum. This is illustrated in Figure 2B. Again, we can solve for the composite effects, and these are shown in Table 1 (column “two environments”). Results show that adding a second environment leaves the dominance and epistatic effects unchanged, confirming that they represent the intrinsic effects of heterozygosity and admixture. Of the remaining quantities, the additive effect, $\left\{\alpha_{1}\right\}$, is now averaged across environments ($r_{1.}^{2}-r_{2.}^{2}\equiv \frac{1}{2}\left(r_{1A}^{2}-r_{2A}^{2}+r_{1B}^{2}-r_{2B}^{2}\right)$), whereas the main environmental effect, {ε}, is simply the difference in fitness between environments, averaged across the parental lines ($r_{.B}^{2}-r_{.A}^{2}\equiv \frac{1}{2}\left(r_{1B}^{2}-r_{1A}^{2}+r_{2B}^{2}-r_{2A}^{2}\right)$).

Finally, the additive‐by‐environment interaction is

(21) $$\begin{array}{cc} \left\{\alpha_{1}\varepsilon \right\} & =\frac{1}{4}\left[\left(r_{1B}^{2}-r_{1A}^{2}\right)-\left(r_{2B}^{2}-r_{2A}^{2}\right)\right] \end{array}$$

(22) $$\begin{array}{cc} & =\frac{1}{2}r_{12}r_{\mathrm{AB}}\rho^{*} \end{array}$$

Here, $-1\leq \rho^{*}\leq 1$ is the cosine similarity of the vector linking the parental phenotypes, and the vector linking the two optima (Fig. 2B): $\left\{\alpha_{1}\varepsilon \right\}$ will be large when the difference between the phenotypes of P1 and P2 resembles the difference between optima A and B. Indeed, the predicted values of $\left\{\alpha_{1}\right\}$, {ε}, and $\left\{\alpha_{1}\varepsilon \right\}$ are equivalent to the quantities described by Blanquart et al. (2013) for measuring local adaptation (see their eqs. 1 and 2), but applied to fitness values that have been transformed and scaled (eq. 6). As shown in Appendix 1 section A1.6 in the Supporting Information, the same framework is easily extendable to other sorts of environmental heterogeneity, namely patchy ecotones, and environmental gradients.

### LOCAL ADAPTATION AND ECOLOGICAL ISOLATION

Although $\left\{\alpha_{1}\varepsilon \right\}$ is a possible measure of local adaptation, it does not describe the extent of ecological isolation between the parental lines. For example, $\left\{\alpha_{1}\varepsilon \right\}$ might be large, even if P1 were fitter than P2 in both habitats (Kawecki and Ebert 2004; Blanquart et al. 2013). However, we do have a measure of isolation in an important special case. If the two parental lines are well adapted to different local optima, then $r_{\mathrm{AB}}^{2}\approx r_{12}^{2}$, and predictions depend on $r_{12}^{2}$ alone. The results with local adaptation are shown in Table 1 (column “local adaptation”).

The effects of varying $r_{12}^{2}$ are illustrated in Figure 3E–G. Each panel shows the expected distance from the optimum, comparing the two parental lines, the initial F1 cross, and the reciprocal backcrosses. The position of the optimum varies across the three panels, and matches (E): the phenotype of P1, (F): the phenotype of P2 and (G): the midparent (i.e., the mean of the P1 and P2 phenotypes). Colors show the effects of varying $r_{12}^{2}$. When $r_{12}^{2}$ is large (red lines), isolation in the parental habitats (Fig. 3E–F) is purely ecological, with P2 and P1 kept distinct solely by environment‐dependent selection against their divergent alleles. In the “intermediate habitat” (Fig. 3G), large $r_{12}^{2}$ leads to hybrid advantage, with all crosses fitter than the parental types (Moore 1977; Yamaguchi and Otto 2020). When $r_{12}^{2}$ becomes small (blue lines), results in all three habitats approach the same W‐shaped pattern of intrinsic isolation, where hybrids beyond the F1 are unfit in all environments (Rundle and Whitlock 2001). By changing the value of $r_{12}^{2}$, we can interpolate between these two extremes.

Taken together, results in Figure 3 show that $r_{12}^{2}$ is both a measure of the amount of meandering in the chain of fixed differences (Fig. 3A–D), and a measure of the relative strengths of ecological isolation versus intrinsic isolation (Fig. 3E–G).

### HYBRID FITNESS ACROSS TIME AND SPACE

**Figure 4:**
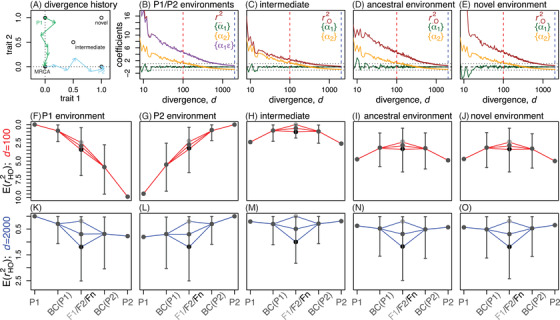
The outcomes of hybridization over time and space. (A) A cartoon of the divergence process that was simulated, with two populations adapting in allopatry to abruptly shifting optima, and then continuing to accumulate divergence via system drift. (B)–(E) change in the composite effects with increasing divergence (Table 1), as measured with respect to (B) both parental environments, or (C)–(E) other single environments. (F)–(O) results for simulated hybrids, plotting $r_{\mathrm{HO}}^{2}$ on a reversed axis, such that fitter genotypes are higher. Points with error bars show the mean and 95% quantiles for 10,000 recombinant hybrids, generated for the reciprocal backcrosses, and the F2. The dark central point (labeled Fn) shows the mean of 10,000 homozygous hybrids, derived from automictic selfing among F1 gametes. Red and blue lines show analytical predictions. These use equation (17), with the measured values of $r_{1O}^{2}$ and $r_{2O}^{2}$, and the assumption that ρ=−1 (for the “intermediate” environment) or ρ=0 (all other cases). Hybrids were scored in the (F)–(J) early stages of divergence (d=100; red lines), and (K)–(O) at later stages (d=2000; blue lines). Simulation procedure is described in Supporting Information Appendix 2, and used the following parameters: N=1000, $N\bar{s}=-10$, 2NU=1, n=2, k=2, free recombination, and “bottom‐up” mutations.

Figure 4 summarizes many of the results above, showing how hybrid fitness varies with genomic composition, environmental conditions, and the amount of divergence. Figure 4 compares analytical results to a single simulation run, which is described in full in Appendix 2 in the Supporting Information. In the simulation, two parental populations adapted to distinct optima in allopatry (shown in cartoon in Fig. 4A). This local adaptation involved fixing ∽50 substitutions, and after this, the populations continued to diverge via system drift. Hybridization between these populations was simulated in five distinct environments, whose optima are also shown in Figure 4A. Figure 4B–E shows the composite effects changing over the complete course of the divergence. In the environments to which the parents are adapted (Fig. 4B), the additive‐by‐environment interaction, $\left\{\alpha_{1}\varepsilon \right\}$ is high in the initial stages of divergence, before declining over time. The same pattern appears, in all environments, with the epistatic effect, $\left\{\alpha_{2}\right\}$ (Fig. 4B–E). This reflects their common dependence on $r_{12}^{2}$ under local adaptation (Table 1). Initially, $r_{12}^{2}$ is high, due to the adaptive phenotypic divergence of the parental lines; then it declines steadily, as stabilizing selection at the new optima is accompanied by genomic evolution via system drift.

The changes in composite effects are reflected in the results for simulated hybrids (Fig. 4F–O). Crosses were formed in the initial stages of divergence (Fig. 4F–J), and at a later stage (Fig. 4K–O). In addition to the standard crosses (also shown in Fig. 3E–G), results are reported for the F2, and an “Fn” cross, derived from automictic selfing among F1 gametes. As such, the three central points in each panel all show balanced hybrids (with $\bar{h}=1/2$), but with maximally different levels of heterozygosity (F1: ${\bar{p}}_{12}=1$; F2: ${\bar{p}}_{12}=\frac{1}{2}$; and Fn: ${\bar{p}}_{12}=0$).

Results after 100 substitutions show the clear signature of ecological isolation in the parental habitats (Fig. 4F–G). In the environment to which P1 is adapted, hybrids tended to be fitter when they carry more P1 alleles, and vice versa (Rundle and Whitlock 2001). For the same reason, in an ecologically intermediate habitat, there was a clear signal of bounded hybrid advantage (Fig. 4H), as hybrids tended to have the favored intermediate phenotype (Moore 1977; Yamaguchi and Otto 2020). Hybrid advantage, at a lower level, also occurred in the ancestral habitat (Fig. 4I); but this had nothing to do with the habitat being ancestral, and the same patterns are observed in an entirely novel habitat, as long as it leads to similar values of $r_{1O}^{2}$ and $r_{2O}^{2}$ (Fig. 4J).

As the genomic divergence increased, and $r_{12}^{2}$ decreased, the outcomes became more and more similar across the environments (Fig. 4K–O). After 2000 substitutions, hybrid fitnesses were already converging toward the characteristic pattern of intrinsic isolation (Fig. 4K–O), with the fixed cost of admixture and the fixed benefit of heterozygosity (eq. 18).

## TWO EXTENSIONS

In this third and final part of the article, we highlight two ways in which the model gives misleading predictions, each resulting from a key simplifying assumption. We then show how these limitations might be overcome.

### LATER CROSSES, AND THE BULMER EFFECT

The project of predicting hybrid fitness solely from the hybrid index and heterozygosity depends on heterospecific alleles appearing in random combinations. This assumption appears to be fairly robust to low recombination (see Supporting Information Appendix 2, Figure A2.9), but nonrandomness can arise for other reasons. Most importantly, selection on the earlier generation hybrids can induce nonrandom associations between alleles in their gametes. This can increase the fitness of later generation hybrids, but without changing allele frequencies in the population as a whole (Bulmer 1971; Walsh and Lynch 2018, chs. 16 & 24). For example, with random union of gametes, the distributions of h and $p_{12}$ will often remain unchanged between the F2 and F3 generations, and so equations (17) and (19) make the same predictions for both. However, selection on the F2 parents can lead to very different levels of fitness.

**Figure 5:**
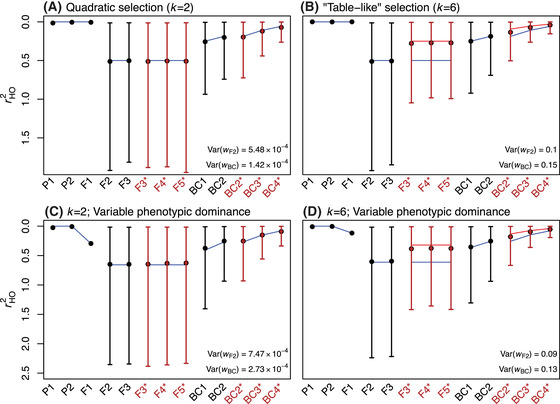
Two extensions to the model, incorporating selection on early‐generation hybrids, and variable phenotypic dominance. Plots show the distance from the optimum, $r_{\mathrm{HO}}^{2}$, on a reverse axis, so that higher points are fitter. After the initial F1 cross, we simulated either random union of gametes among the hybrids (F2–F5), or repeated backcrossing to parental line P1 (BC1–BC4). For the later crosses, we chose parents either wholly at random (black points and lines), or with a probability proportional to their fitness (asterisks and red points and lines). In each case, results for 10,000 simulated hybrids (mean and 95% quantiles), are compared to analytical predictions. Blue lines show predictions that ignore the effects of selection on earlier hybrids (eq. 24 with β=0); red lines show predictions assuming that only optimally‐fit parents reproduce (eq. 24 with β=1/2). (A) results with quadratic selection (eq. 3 with k=2) such that there is limited variation in parental fitness. (B) results with truncation‐like selection (eq. 3 with k=6), and high variance in parental fitness. (C) and (D) equivalent results, when populations were simulated with variable phenotypic dominance (such that heterozygous effect of each new mutation was the homozygous effect, multiplied by a uniformly distributed random variable). The clearest consequence is that the F1 are suboptimal, even when the parental lines are optimal. Here, predictions use equations (27) and (28) with the observed $r_{\mathrm{GO}}^{2}$, and v=1/12, from the variance of a uniform distribution. All predictions assumed optimally‐fit parents ($r_{.O}^{2}=0$). Simulations are described in Supporting Information Appendix 2, and used the following parameters: N=1000, $N\bar{s}=-0.1$, 2NU=1, n=2, $\bar{c}=0.5$, “bottom‐up” mutations, stationary optima matching the ancestral state; hybrids were formed as soon as one of the diverging populations had fixed 1000 substitutions.

To see this, let us consider the case of free recombination ($\bar{c}=1/2$), and optimal parental lines ($r_{.O}^{2}=0$). With these assumptions, the variance in trait values (the $z_{i}$) among F3 offspring is

(23) $$\mathrm{Var}\left(z_{i,F3}\right)=\frac{1}{2}\left[\mathrm{Var}\left(z_{i,F2}\right)+\mathrm{Var}\left(z_{i,F2\left(\mathrm{sel}\right)}\right)\right]\;\bar{c}=1/2,r_{.O}^{2}=0$$

where $z_{i,F2}$ and $z_{i,F2(sel)}$ are the trait values for the total F2 population, and for the subset of selected parents (see Walsh and Lynch 2018, ch. 16, assuming complete heritability). In the extreme case, if only optimal F2 reproduce, then $\mathrm{Var}(z_{i,F2(sel)})=0$ and $\mathrm{Var}\left(z_{i,F3}\right)=\frac{1}{2}\mathrm{Var}\left(z_{i,F2}\right)$. In general, the expected distance to the optimum of later generated hybrids can be written as

(24) $$E\left(r_{\mathrm{HO}\left(\text{sel}\right)}^{2}\right)=E\left(r_{\mathrm{HO}}^{2}\right)\left(1-\beta \right)\;\bar{c}=1/2,r_{.O}^{2}=0$$

where $E(r_{\mathrm{HO}}^{2})$ is the prediction of equation (18), which neglects the effects of selection on earlier‐generation hybrids. The parameter 0≤β≤1/2 captures the effects of this selection, reaching its upper bound when only optimal individuals reproduce. This is illustrated in Figure 5A and B. We chose two simulation runs where populations diverged despite a fixed optimum, via system drift. If we generated an F3 cross using a random selection of F2 parents, then equation (18) applies well (see blue lines in Fig. 5). If we selected parents with a probability proportional to their fitness (eq. 3), the same results continued to apply for later crosses, but only when the fitness function was quadratic, that is, when we set k=2 in equation (3). In this case, there was very little inter‐individual variation in fitness. The results, shown in Figure 5A, imply that β≈0 with quadratic selection. Results with k=6 are shown in Figure 5B. Setting k=6 in equation (3) generates a “table‐like” fitness function (Fraïsse et al. 2016), equivalent to strong truncation selection, and this generates high variation in parental fitness. In this case, results were close to the lower bound of equation (24), such that β≈1/2 (see red lines in Fig. 5B). The same patterns continued unchanged for other late generation crosses, including the F4 and F5, and also applied to repeated backcrosses to the P1 line (Fig. 5A–B).

### PHENOTYPIC DOMINANCE

The most implausible prediction of Fisher's model is embodied in equation (18). This equation predicts that, at very high levels of divergence, fully heterozygous hybrids will always be as fit as their parents (this is because, when $p_{12}=1$ and h=1/2, the benefits of heterozygosity exactly cancel the costs of admixture; Barton 2001; Fraïsse et al. 2016; Schiffman and Ralph 2017).

This prediction is implausible because—with strictly biparental inheritance and expression—the initial F1 cross will be globally heterozygous (i.e., will carry one allele from each parent at all d divergent loci). Although many intrinsically isolated species do produce fit F1 (Wallace 1991; Price and Bouvier 2002; Fraïsse et al. 2016), F1 fitness tends to decline as the parents become very genetically divergent, even in environments where both parents are well adapted (Endler 1977; Bateson 1978; Waser 1993; Price and Bouvier 2002; Edmands 2002; Fraïsse et al. 2016). The model also struggles to explain a second widespread pattern in the F1: when some loci have uniparental inheritance or expression, the reciprocal F1 often have very different fitnesses, even if the parents are both well adapted (Bolnick and Near 2005; Turelli and Moyle 2007; Escobar et al. 2008; Brandvain et al. 2014; Sato et al. 2014; Fraïsse et al. 2016; Bouchemousse et al. 2016). Fisher's model can only account for these asymmetries if the globally heterozygous genotype is suboptimal (see Fraïsse et al. 2016 for details).

In this section, we show that these features of Fisher's model result from phenotypic additivity, and are improved by adding phenotypic dominance (Manna et al. 2011). To see this, let us replace equation (5) with

(25) $$z_{i}=z_{P1,i}+\sum_{j\in J_{\text{hom}}}m_{\mathrm{ij}}+\sum_{j\in J_{\text{het}}}\left(\frac{1}{2}m_{\mathrm{ij}}+\delta_{\mathrm{ij}}\right)$$

where $\delta_{ij}$ is the deviation from semi‐dominance on trait i, caused by introducing substitution j in heterozygous form. We now assume that the $\delta_{ij}$ can be treated as the increments of a new, and independent Brownian bridge, linking the midparental value of trait i, to the trait value of the global heterozygote (see the phenotypes labeled P and G in Fig. 2C). (We note that this strong assumption does not allow for differences in the typical dominance relations of large‐ and small‐effect changes; Wright 1929; Manna et al. 2011; Fraïsse et al. 2016). In Appendix 1 section A1.4 in the Supporting Information, we show that these assumptions lead to

(26) $$\begin{array}{cc} E\left(\left(r_{\mathrm{HO}}^{2}\right|h,p_{12}\right) & \approx V+M+V_{\delta }+M_{\delta } \end{array}$$

where V and M are the additive results from equations (11)–(14), and $V_{\delta }$ and $M_{\delta }$ are the new contributions from variable dominance. These new contributions are

(27) $$\begin{array}{cc} V_{\delta }= & \;4vp_{12}\left(1-p_{12}\right) \end{array}$$

(28) $$\begin{array}{cc} M_{\delta }= & \;p_{12}\left(r_{\mathrm{GO}}^{2}-r_{\mathrm{PO}}^{2}\right)\\ & -p_{12}\left(1-p_{12}\right)r_{\mathrm{PG}}^{2}\\ & -p_{12}\left(h-\frac{1}{2}\right)\left(r_{2G}^{2}-r_{1G}^{2}\right) \end{array}$$

(Supporting Information Appendix 1 section A1.4). In equation (27), a new parameter, v, describes the scaled variance of the $\delta_{ij}$. Equation (28) depends on several new distances, and these are illustrated in Figure 2C. The corresponding changes in the composite effects are listed in Table 1 (column “phenotypic dominance”). Table 1 shows that phenotypic dominance adds two new composite effects: $\left\{\delta_{2}\right\}$ and $\left\{\alpha_{1}\delta_{1}\right\}$, and alters the value of a third, $\left\{\delta_{1}\right\}$, so that it is no longer a constant.

Equations (27) and (28) are both proportional to $p_{12}$ and so they alter predictions only for heterozygous hybrids. The predictions are altered in two major ways. First, a nonzero value of $\left\{\alpha_{1}\delta_{1}\right\}$ (which corresponds to the third term in eq. 28), now allows for “directional dominance.” For example, in Figure 2C, the global heterozygote, G, is much closer to the P2 phenotype than to the P1 phenotype ($r_{2G}^{2}<r_{1G}^{2}$), which implies that P2 alleles are dominant on average. This sort of asymmetry allows Fisher's model to account for “dominance drive” in hybrid zones, where alleles can spread due to their dominance relations alone (Mallet and Barton 1989; Barton 1992). Second, when the phenotype of the global heterozygote, G, differs from the midparental phenotype, P, the effects of heterozygosity are qualitatively altered. Under the additive model, these effects are intrinsic and always beneficial (see eq. 17). By contrast, with phenotypic dominance, the effects of heterozygosity become extrinsic, and so they can vary over time and space. Furthermore, at large divergences, heterozygosity will tend to become deleterious. This is because the global heterozygote—unlike the parental genotypes—may never be exposed to natural selection, and cannot, in any case, breed true. As such, its phenotype can continue to wander away from the optimum as divergence increases, even if effective stabilizing selection acts on the parental phenotypes. The result is that the distance $r_{\mathrm{GO}}^{2}$, unlike $r_{\mathrm{PO}}^{2}$, has no tendency to vanish as divergence increases.

In Appendix 2 in the Supporting Information, we report a full set of individual‐based simulations, incorporating variable dominance, which support the analytical results above. The most important consequence of variable dominance is illustrated in Figure 5C and D. Here, most results closely match those of the additive model, as shown in Figure 5A and B. The exception is the F1, which with dominance, is noticeably less fit than the parental lines (Fig. 5C and D).

## Discussion

### FISHER'S MODEL AS A FITNESS LANDSCAPE

Using fitness landscapes based on Fisher's geometric model, we have developed analytical predictions for the fitness of hybrids between divergent lines. These predictions allowed us explore several factors that can affect hybrid fitness. These factors are (i) the genotypic composition of the hybrids—parameterized by the hybrid index, h, and heterozygosity $p_{12}$; (ii) the environmental conditions—parameterized by $r_{1O}^{2}$ and $r_{2O}^{2}$: the distance to the current optimum of the parental lines; and (iii) the divergence history of the populations—parameterized by $r_{12}^{2}$: the distance between the parental phenotypes (Fig. 2A). We have also shown how these distances tend to change over the course of evolutionary divergence. Because our results apply to wide range of evolutionary and ecological scenarios, we can classify scenarios according to the values of a small number of geometric distances.

Of course, the simplicity of the results stems from the simplicity of the fitness landscape, and so we have to ask whether the model is overly simple. This question is not settled by pointing to the toy nature of the phenotypic model (optimizing selection on n quantitative traits). This model is best viewed as approximating more complex and realistic phenotypic models (Martin 2014; Schiffman and Ralph 2017; Fraïsse and Welch 2019); and these approximations can involve many‐to‐one mappings, so that, in principle, any n‐dimensional phenotype under Fisher's model, could correspond to multiple real‐world phenotypes. As such, the model has to be judged by its successes and failures in accounting for observed patterns in hybrid fitness (Fraïsse et al. 2016; Simon et al. 2018).

In cases where Fisher's model is inadequate, we have also shown how it can be extended. In particular, adding variable phenotypic dominance allows for a low fitness F1 between highly divergent, but equally fit parental lines (Fraïsse et al. 2016; Fig. 5C and D). This extension further supports previous claims that Fisher's model can incorporate other modeling approaches as special cases (Simon et al. 2018). For example, when the parental lines have high fitness, equation (26) gives identical predictions to a model of Dobzhansky‐Muller incompatibilities, with variable dominance relations (Turelli and Orr (2000); see eq. A37 of Simon et al. 2018).

### ESTIMATING THE KEY QUANTITIES

By connecting Fisher's model to the quantitative genetics of line crosses (Hill 1982; Lynch 1991; Rundle and Whitlock 2001; Demuth and Wade 2005; Yamaguchi and Otto 2020), we have shown that the geometric distances are closely related to the composite effects. This implies that the distances can be estimated using measurements of fitness, or some component of fitness, taken from controlled crosses (Lynch and Walsh 1998; Lynch 1991; Rundle and Whitlock 2001; Simon et al. 2018). This claim comes with an important caveat. The results in Table 1 apply not to raw fitness values, but to values that have been suitably transformed and scaled. In our notation, they apply to $r_{\mathrm{HO}}^{2}$ and not to w (see eqs. (3), (4), (5), (6)). Data transforms are an inherent part of quantitative genetics (Lynch and Walsh 1998, ch. 11), but there is also the need to estimate the scaling factor in equation (6). This extra parameter is relatively easy to estimate from a diverse collection of hybrids (see Simon et al. 2018), but not from a limited number of controlled crosses (Yamaguchi and Otto 2020). We ducked this issue in Figure 4, by estimating equation (7) directly from the simulated fixed effects (see Supporting Information Appendix 1 section A1.1). This is a real limitation, but there are many special cases where the distances can be estimated from fitness values that are transformed but unscaled (i.e., from the numerator of eq. 6). For example, with two locally adapted populations (Table 1), the distance $r_{12}^{2}$ can be estimated from a ratio of composite effects:

(29) $$\hat{r_{12}^{2}}=\frac{4\left\{\alpha_{1}\varepsilon \right\}}{\left\{\alpha_{1}\varepsilon \right\}-2\left\{\alpha_{2}\right\}}$$

so that the scaling factor cancels. Simon et al. (2018) give other, similar examples.

These methods of estimation all assume that hybrid fitness can be meaningfully predicted from the hybrid index and heterozygosity alone. However, we have also shown that this assumption breaks down when there are strong disequilibria between heterospecific alleles, as generated by selection on early generation hybrids (as opposed to selection during the divergence). These effects are weak in some parameter regimes (Fig. 5A), but observations of strong incompatibilities, involving small genomic regions, suggests that they might be important in nature (Barton 2001; Coyne and Orr 2004, ch. 8; Fraïsse et al. 2016). We have provided a simple solution, which applies with strong, truncation‐like selection (eq. 24; Fig. 5B). However, this approach might be difficult to apply to entire hybrid swarms, when some, but not all hybrids have strong selectively induced disequilibria (Jiggins and Mallet 2000; Allendorf et al. 2001; Vernesi et al. 2003; Simon et al. 2018). Even greater challenges will arise when selection changes allele frequencies (Walsh and Lynch 2018, chs. 16 & 24). In both cases, the distributions of h and $p_{12}$ will not be sufficient to predict hybrid fitnesses.

### THE PROCESS OF DIVERGENCE AND THE OUTCOME OF HYBRIDIZATION

Because it is a well‐studied model of evolutionary divergence, Fisher's model is especially useful for investigating the connections between the mode of divergence between the parental lines, and the outcome of hybridization between them.

One set of connections has been explored extensively in previous work. Compared to drift, positive selection will lead to divergence that is more rapid and more resistant to the swamping effects of gene flow, and tend to fix effects that are larger and more variable in size (Orr 1998b; Griswold 2006; Yeaman and Whitlock 2011; Rockman 2012; Matuszewski et al. 2014; Débarre et al. 2015; Matuszewski et al. 2015; Dittmar et al. 2016; Thompson et al. 2019; Yamaguchi and Otto 2020). And larger changes will often have a greater influence on hybrid fitness (Chevin et al. 2014; Fraïsse et al. 2016; Yamaguchi and Otto 2020). Together, these facts will tend to implicate natural selection, rather than drift, in any hybrid problems that appear early in the divergence process (Jiggins and Mallet 2000; Coyne and Orr 2004, ch. 11; Yamaguchi and Otto 2020). In the results presented here, these effects of selection are all incorporated into the scaling factor (eqs. 6 and 7), with positive selection tending to lead to larger values of d and larger values of the $\lambda_{i}$.

We have focused on a different set of connections between divergence and hybridization, and these are captured by the distance $r_{12}^{2}$. This distance can be called “intrinsic,” because it is a property of the parental lines, which does not depend on the current position of the optimum. For this reason, $r_{12}^{2}$ describes the possible outcomes of hybridization in a variety of environmental conditions (Figs. 3E–G and 4). For example, when parental lines are well adapted to different habitats, a high value of $r_{12}^{2}$ implies that the isolation between the lines will be purely ecological. In an intermediate habitat, or wherever the parents are poorly adapted, lines with a high $r_{12}^{2}$ are more likely to generate hybrid advantage beyond the F1. In this way, the value of $r_{12}^{2}$ is closely related to the notion of “coadaptation” among the parental alleles (Wallace 1991). This is also why $r_{12}^{2}$ determines $\left\{\alpha_{2}\right\}$: the additive‐by‐additive epistatic effect (Table 1; Lynch 1991).

As well as describing the outcomes of hybridization, $r_{12}^{2}$ contains some information about the mode of divergence. This information is not about epistasis: the value of $\left\{\alpha_{2}\right\}$ tells us nothing at all about the role of epistatic genetic variance during divergence (Lynch 1991; Welch 2004; Demuth and Wade 2005; Barton 2017; see Supporting Information Appendix 2). Instead, we have shown that $r_{12}^{2}$ measures the exchangeability of divergent alleles, or—equivalently—the consistency in their “directions” in trait space, and compares this consistency to expectations under a random walk. As such, high values of $r_{12}^{2}$ imply that line‐specific alleles are more similar to each other than expected (Fig. 3A–D). This definition shows that $r_{12}^{2}$ is closely connected to standard tests for natural selection on quantitative traits, such as the QTL sign test (Orr 1998a), or the Qst‐Fst comparison (Spitze 1993; Whitlock and Guillaume 2009). Indeed, simulations confirm that adaptive divergence, especially in parapatry, is most likely to lead to high values of $r_{12}^{2}$ (see Supporting Information Appendix 2, section A2.2).

Together, these results clarify what hybrid fitness can and cannot tell us about the mode of parental divergence. On one hand, some patterns of hybrid fitness—those associated with high $r_{12}^{2}$—are reliable indicators of selectively driven divergence, and especially of local adaptation maintained in the face of gene flow. On the other hand, patterns associated with low $r_{12}^{2}$ can arise in a variety of ways, including via adaptive divergence, especially in allopatry (e.g., Fig. 3D). (These limitations are closely related to the low power of the QTL sign test: Rice and Townsend 2012; Walsh and Lynch 2018, ch. 12). Furthermore, unless there is substantial gene flow, any signature of selection will be transient. Over time, the model predicts convergence to an identical pattern of intrinsic reproductive isolation, whatever the mode of divergence (Figs. 3 and 4).

## AUTHOR CONTRIBUTIONS

JW, NB, and HS designed the project. JW, BDS, and HS performed the analysis. HS, DR, and JW wrote the simulation code and ran the simulations. JW, HS, and BDS wrote the manuscript, with contributions from all authors. HS and BDS equally contributed to this study.

## DATA ARCHIVING

Simulation code and simulated data are deposited on Dryad (https://doi.org/10.5061/dryad.hmgqnk9f9).

Associate Editor: C. Bank

Handling Editor: D. Hall

## Supporting information


**Table A2.1**: Population genetic parameter values used in the simulations.
**Figure A2.1**: Simulated evolution of a single population with a stationary phenotypic optimum, and a stable population size of *N* = 1000.
**Figure A2.2**: Simulated evolution of single populations under demographic and environmental change.
**Figure A2.3**: Illustrations of the 15 divergence scenarios that were simulated.
**Figure A2.4**: Simulated divergence between pairs of populations.
**Figure A2.5**: The robustness of the Brownian bridge approximation.
**Figure A2.6**: Evidence for deviations from multivariate normality in the fixed effects, that differentiate the simulated populations.
**Figure A2.7**: Simulated crosses in different environments.
**Figure A2.8**: Simulated crosses when parental populations diverged via drift, using divergence scenarios 11‐12 in Figure A2.4.
**Figure A2.9**: Simulated crosses after local adaptation, in populations with low recombination.
**Figure A2.10**: The Brownian bridge approximation with and without variable phenotypic dominance.
**Figure A2.11**: The Brownian bridge approximation for heterozygous hybrids, with variable phenotypic dominance.
**Figure A2.12**: The second term of the Brownian bridge approximation, with and without phenotypic dominance.
**Figure A2.13**: With variable phenotypic dominance, the phenotype of the global heterozygote, G, is usually further from the optimum than the midparental phenotype, P.
**Figure A2.14**: Simulated hybridization after local adaptation, with variable phenotypic dominance.
