# Supplementary material for: The geometry and genetics of hybridization
Source: Evolution. 2020 Nov 23;74(12):2575–90. doi: 10.1111/evo.14116 (PMC7839769; doi:10.1111/evo.14116)
Supplement: Supplementary file 1 — Table A2.1: Population genetic parameter values used in the simulations. Figure A2.1: Simulated evolution of a single population with a stationary phenotypic optimum, and a stable population size of N = 1000. Figure A2.2: Simulated evolution of single populations under demographic and environmental change. Figure A2.3: Illustrations of the 15 divergence scenarios that were simulated. Figure A2.4: Simulated divergence between pairs of populations. Figure A2.5: The robustness of the Brownian bridge approximation. Figure A2.6: Evidence for deviations from multivariate normality in the fixed effects, that differentiate the simulated populations. Figure A2.7: Simulated crosses in different environments. Figure A2.8: Simulated crosses when parental populations diverged via drift, using divergence scenarios 11‐12 in Figure A2.4. Figure A2.9: Simulated crosses after local adaptation, in populations with low recombination. Figure A2.10: The Brownian bridge approximation with and without variable phenotypic dominance. Figure A2.11: The Brownian bridge approximation for heterozygous hybrids, with variable phenotypic dominance. Figure A2.12: The second term of the Brownian bridge approximation, with and without phenotypic dominance. Figure A2.13: With variable phenotypic dominance, the phenotype of the global heterozygote, G, is usually further from the optimum than the midparental phenotype, P. Figure A2.14: Simulated hybridization after local adaptation, with variable phenotypic dominance. [file EVO-74-2575-s001.pdf]

Appendix 1: Derivations

In this Appendix, we present derivations for the major results in the main text.

A1.1 Removing correlations in the fitness function and fixed factors

To derive the Brownian bridge approximation for Fisher's geometric model (see section A1.2 below), we assume that the fixed effects on each trait are uncorrelated, and will also assume uncorrelated selection on each trait, as shown in eq. 2. In this section, we show that these results apply more generally, as long as the distribution of fixed effects is sufficiently close to multi-variate normality, potentially involving correlations among traits, and as long as the distance metric (eq. 2) is a multi-variate Gaussian, potentially involving correlated selection (Waxman and Welch, 2005). This is achieved by transforming the phenotypic space. This "simultaneous diagonalization", allows us to work with a far simpler class of models, without any loss of generality. The treatment below is very similar to that of Martin and Lenormand (2006a), and follows most notation from Mathai and Provost (1992).

We start with a vector X of phenotypic effects in an n-dimensional space, which follows a multivariate normal distribution, that is,

$$X \sim N(\mu, \Sigma) \tag{30}$$

We also have an arbitrary quadratic form

$$Q(X) = X^T A X \tag{31}$$

where A is positive definite. In the model, $Q(X)$ functions as a general formula for the distance to the optimum, which, without loss of generality, is assumed to be at the origin. In particular, as in Martin and Lenormand (2006a), the diagonal entries of A are the selective effects of each trait, and off-diagonal entries correspond to selective interactions between traits. The positive definiteness of A means that we cannot attain a negative value for $S(X)$, so that fitness is maximized at the optimum.

Now define

$$Y = \Sigma^{-1/2}(X - \mu) \tag{32}$$

so that $Y \sim N(0,I)$. Then diagonalize the matrix $\Sigma^{1/2}A\Sigma^{1/2}$ as

$$P^T\Sigma^{1/2}A\Sigma^{1/2}P = \Lambda \tag{33}$$

where P is an orthogonal matrix so that $P^TP = I$ and Λ is a diagonal matrix of eigenvalues of ΣA. Now let

$$U = P^TY \tag{34}$$

so that $Y = PU$ and $U \sim N(0,I)$. Now we have

$$Q(X) = X^TAX = (Y + \Sigma^{-1/2}\mu)^T\Sigma^{1/2}A\Sigma^{1/2}(Y + \Sigma^{-1/2}\mu) = (U+b)^T\Lambda(U+b) \tag{35}$$

where $b = (P^T\Sigma^{-1/2}\mu)^T$. Lastly, defining $Z = U + b$, we have

$$Q(X) = X^TAX = Z^T\Lambda Z = \sum_{j=1}^{n}\lambda_jZ_j^2 \tag{36}$$

where $Z \sim N(b,I)$ is our transformed phenotypic vector with no correlation between traits, Λ is our transformed, diagonal selection matrix. This procedure leads to the distance measure of eq. 2, and explains our use of isotropic selection in the simulations. Of course, in practice, we are unlikely to know the exact distribution of the phenotypic effects, and even from simulations, we can only measure their sample mean and covariance. This means that the normalizing constant in eq. 6 has to be estimated in other ways (see Discussion).

A1.2 The Brownian bridge approximation

In this section, we derive eqs. 9-13. Equivalent results were also presented by Simon et al. (2018), but using a different method. Having defined the independent traits (see section A1.1 above), we start with eqs. 4-5, and then assume that random collections of heterospecific alleles describe a Brownian bridge on each trait. We do this by treating the homozygous effects of the P2 alleles (i.e., the m_{ij}), as increments in the bridge, linking the trait values of P1 and P2. We note here that the factor 4 in eq. 6 appears for consistency with Simon et al. (2018), who considered a bridge applying to heterozygous alleles, and

ending at the midparent; $E\left(r_{\mathrm{HO}}^2 \middle| h, p_{12}\right) \equiv f$ in their notation. From the standard properties of Brownian bridges (Revuz and Yor, 1999), we have:

$$E\left(m_{ij}\right) = \frac{z_{\mathrm{P2},i} - z_{\mathrm{P1},i}}{d} \tag{37}$$

$$\mathrm{Cov}\left(m_{ij}, m_{ab}\right) = \begin{cases} 0, & i \neq a \\ 1 - 1/d, & i = a, j = b \\ -1/d, & i = a, j \neq b \end{cases} \tag{38}$$

(where here, and below, all expectations are over the set of d substitution effects on a single trait, i). Using eqs. 37-38 and eq. 5, we can now derive the mean and variance of the value of trait i.

$$\begin{aligned} E\left(z_i - o_i\right) &= z_{\mathrm{P1},i} - o_i + E\left(\sum_{j \in J_{hom}} m_{ij}\right) + \frac{1}{2} E\left(\sum_{j \in J_{het}} m_{ij}\right) \\ &= z_{\mathrm{P1},i} - o_i + d p_2 E\left(m_{ij}\right) + d p_{12} \frac{1}{2} E\left(m_{ij}\right) \\ &= z_{\mathrm{P1},i} - o_i + d\left(p_2 + \frac{p_{12}}{2}\right) \frac{z_{\mathrm{P2},i} - z_{\mathrm{P1},i}}{d} \\ &= (1-h)(z_{\mathrm{P1},i} - o_i) + h(z_{\mathrm{P2},i} - o_i) \end{aligned} \tag{39}$$

Similarly, for the variance, we find:

$$\begin{aligned}\mathrm{Var}\left(z_i - o_i\right) = \mathrm{Var}\left(z_i\right) =&\mathrm{Var}\left(\sum_{j\in J_{hom}} m_{ij}\right)\\ &+\mathrm{Var}\left(\sum_{j\in J_{het}} \frac{1}{2} m_{ij}\right)\\ &+2\mathrm{Cov}\left(\sum_{j\in J_{hom}} m_{ij}, \frac{1}{2}\sum_{b\in J_{het}} m_{ib}\right)\end{aligned}$$

$$\begin{aligned}=&dp_2\mathrm{Var}\left(m_{ij}\right) + dp_2(dp_2-1)\mathrm{Cov}\left(m_{ij}, m_{ib}\right)\\ &+dp_{12}\mathrm{Var}\left(\frac{1}{2}m_{ij}\right) + dp_{12}(dp_{12}-1)\mathrm{Cov}\left(\frac{1}{2}m_{ij}, \frac{1}{2}m_{ib}\right)\\ &+2dp_2 dp_{12}\mathrm{Cov}\left(\frac{1}{2}m_{ij}, m_{ib}\right)\end{aligned}$$

$$\begin{aligned}=&dp_2(1-p_2)\\ &+dp_{12}(1-p_{12})\frac{1}{4}\\ &-dp_2 p_{12}\end{aligned}$$

$$=d\left[h(1-h) - \frac{p_{12}}{4}\right] \tag{40}$$

The key results of eqs. 11-14, now follow from combining eqs. 39 and 40 with the definition of the distance metric (eq. 2), and the scaling factor (eq. 6). Equation 13 also uses the definition of cosine similarity:

$$\rho = \frac{\langle \mathbf{z}_{\mathrm{P1}} - \mathbf{o}, \mathbf{z}_{\mathrm{P2}} - \mathbf{o}\rangle_\lambda}{\|\mathbf{z}_{\mathrm{P1}} - \mathbf{o}\|_\lambda \|\mathbf{z}_{\mathrm{P2}} - \mathbf{o}\|_\lambda} \equiv \frac{\sum \lambda_i (z_{\mathrm{P1},i} - o_i)(z_{\mathrm{P2},i} - o_i)}{\sqrt{\sum \lambda_i (z_{\mathrm{P1},i} - o_i)^2}\sqrt{\sum \lambda_i (z_{\mathrm{P2},i} - o_i)^2}} \tag{41}$$

A1.3 The scaled standard deviation in the distance to the optimum

Our main results apply only to the mean distance to the optimum, across a set of hybrids. However, we can also derive a comparable result for its standard deviation. This result depends much more strongly on the (assumed) normality of the trait values, z_i, and so a failure of its predictions is indicative of non-normal effects. Under normality, we have

$$\mathrm{Var}\left(r_{\mathrm{HO}}^2 \middle| h, p_{12}\right) \approx \frac{16}{d^2\left(\sum_i \lambda_i\right)^2} 2\sum_i \lambda_i^2 \mathrm{Var}\left(z_i\right)\left(\mathrm{Var}\left(z_i\right) + 2E^2\left(z_i - o_i\right)\right) \tag{42}$$

which yields the following prediction for fully homozygous genotypes:

$$\sqrt{\mathrm{Var}\left(r_{\mathrm{HO}}^2 \middle| h, p_{12}\right) \frac{\left(\sum_i \lambda_i\right)^2}{32\sum_j \lambda_j^2} - 2h(1-h)\frac{\sum_i \lambda_i E^2\left(z_i - o_i\right)}{d^2 \sum_j \lambda_j^2}} = 4h(1-h), \qquad p_{12} = 0 \tag{43}$$

This quantity is plotted for our individual-based simulations in Appendix 2, Figure A2.6. Its poor fit to predictions, especially for adaptive divergence, indicates deviations from normality in the simulated fixed effects.

A1.4 Results with variable phenotypic dominance

To derive eqs. 25-28 - the results with variable phenotypic dominance - we use the approach of section A1.2, but applied to the dominance effects (the δ_{ij}) instead of the homozygous effects (the m_{ij}). The result for V_δ (eq. 27) follows directly from the (strong) assumption that the δ_{ij} show no covariance between traits, or with the m_{ij}. As shown in Appendix 2, Figure A2.11, this assumption did not hold in a small number of our simulation runs. The violation of the assumptions was evident in the negative estimates of the parameter v, which appears in eq. 27 and parameterizes the variance in the δ_{ij}.

To derive eq. 28, we note that the mean trait values are affected by an extra term, describing the

Brownian bridge between the global heterozygote (denoted G in Figure 2c) and the midparent (denoted P in Figure 2c). As such, eq. 39 is replaced by

$$E\left(z_i - o_i\right) = z_{\mathrm{P1},i} - o_i + p_2(z_{\mathrm{P2},i} - z_{\mathrm{P1},i}) + p_{12}\left(z_{\mathrm{G},i} - z_{\mathrm{P1},i}\right)$$

$$= z_{\mathrm{P1},i} - o_i + h(z_{\mathrm{P2},i} - z_{\mathrm{P1},i}) + p_{12}\left(z_{\mathrm{G},i} - \frac{z_{\mathrm{P1},i} + z_{\mathrm{P2},i}}{2}\right)$$

and so, with $z_{\mathrm{P},i} = (z_{\mathrm{P1},i} + z_{\mathrm{P2},i})/2$ denoting the midparental trait value, the squared mean is:

$$\begin{aligned} E^2\left(z_i - o_i\right) = & \left(z_{\mathrm{P1},i} - o_i\right)^2 + h^2\left(z_{\mathrm{P2},i} - z_{\mathrm{P1},i}\right)^2 + p_{12}^2\left(z_{\mathrm{G},i} - z_{\mathrm{P},i}\right)^2 \\ & + 2h\left(z_{\mathrm{P1},i} - o_i\right)\left(z_{\mathrm{P2},i} - z_{\mathrm{P1},i}\right) \\ & + 2p_{12}\left(z_{\mathrm{P1},i} - o_i\right)\left(z_{\mathrm{G},i} - z_{\mathrm{P},i}\right) \\ & + 2hp_{12}\left(z_{\mathrm{P2},i} - z_{\mathrm{P1},i}\right)\left(z_{\mathrm{G},i} - z_{\mathrm{P},i}\right) \end{aligned}$$

After applying the scaling of eq. 6, and the cosine rule, this reduces to $M + M_\delta$, as given in eqs. 14 and 28.

A1.5 The distance between the parental phenotypes

In this section, we describe r_{12}^2; this is the distance between the parental phenotypes as shown in Figure 2a, and defined in eq. 15. We derive the maximum value of r_{12}^2, and show how its value relates to a random walk in phenotypic space, and to the exchangeability of the alleles that are specific to each parental line.

First, as above, we denote as $\mathbf{m}_i$ the n-dimensional vector of phenotypic change caused by the ith substitution differentiating the parental lines (recalling that substitutions are defined relative the P1 parental genotype; see Figures 1 and 3). The magnitude of $\mathbf{m}_i$ is

$$\|\mathbf{m}_i\|_\lambda \equiv \sqrt{\sum_{j=1}^{n} \lambda_j m_{ji}^2} \tag{44}$$

The numerator of eq. 15, which is the squared Euclidean distance between the parental phenotypes, is equal to the squared magnitude of the sum of the $\mathbf{m}_i$.

$$\|\mathbf{z}_{\mathrm{P2}} - \mathbf{z}_{\mathrm{P1}}\|_\lambda^2 = \left\| \sum_{i=1}^{d} \mathbf{m}_i \right\|_\lambda^2 \tag{45}$$

The denominator of eq. 15 involves the scaling factor of eq. 6. We now show that this denominator is equal to the expected length of a random walk in phenotype space, conditioned on the observed distribution of effect sizes. Under a random walk, the vectors will be orthogonal on average, and so, given the way that the λ_i are defined (Appendix 1 section A1.1), this distance is equal to the sum of the squared magnitudes.

$$d\,\|\mathbf{1}\|_\lambda^2 = \sum_{i=1}^{d} \|\mathbf{m}_i\|_\lambda^2$$

$$= d\left[\mathrm{Var}\left(\|\mathbf{m}_i\|_\lambda\right) + E^2\left(\|\mathbf{m}_i\|_\lambda\right)\right] \tag{46}$$

Finally, we note that the length of a sum of vectors is maximized when they all point in the same direction, so that the magnitude of the sum is the sum of the magnitudes. In this case, the maximized squared magnitude is

$$\max\left(\left\| \sum_{i=1}^{d} \mathbf{m}_i \right\|_\lambda^2\right) = \left(\sum_{i=1}^{d} \|\mathbf{m}_i\|_\lambda\right)^2$$

$$= d^2 E^2\left(\|\mathbf{m}_i\|_\lambda\right)$$

Putting these together, we have

$$\max\left(r_{12}^2\right) = 4\frac{\max\left(\|\sum_{i=1}^d \mathbf{m}_i\|_\lambda^2\right)}{\sum_{i=1}^d \|\mathbf{m}_i\|_\lambda^2}$$

$$= \frac{4d}{1+CV^2(\|\mathbf{m}_i\|_\lambda)} \tag{47}$$

where $CV(\|\mathbf{m}_i\|_\lambda) = \mathrm{Std}(\|\mathbf{m}_i\|_\lambda)/E(\|\mathbf{m}_i\|_\lambda)$ is the coefficient of variation in the magnitudes. When populations adapt to a distant stationary optimum via new mutations of variable sizes, (Orr, 1998b) showed that the $\|\mathbf{m}_i\|$ are often approximately exponentially distributed. In this case, $CV(\|\mathbf{m}_i\|_\lambda) \approx 1$, so that $\max\left(r_{12}^2\right) \approx 2d$. The absolute maximum of $4d$ occurs when all vectors are of equal magnitude, such that $CV(\|\mathbf{m}_i\|_\lambda) = 0$ and all P2 alleles are fully exchangeable. This result also follows from the triangle inequality.

A1.6 Hybrid fitness in patchy ecotones and environmental gradients

In this section, we briefly extend results for Fisher's model with two optima, to encompass two other sorts of environmental heterogeneity, These are (1) hybrids scored in patchy ecotones, where each individual experiences one of two alternative habitat types, with some fixed probability; and (2) hybrids scored along an environmental gradient, such that the two optimal values represent the extremes of a continuum.

First, let us consider the patchy ecotone, and assume that hybrids experience habitat-type B with probability q, and habitat-type A with probability $1-q$. We begin by adding the environmental indicator variable of Rundle and Whitlock (2001) to eqs. 9-14. Recall that $\theta_E \equiv 2I-1$, where $I = 0$ for individuals experiencing habitat-type A, and $I = 1$ for individuals experiencing in habitat-type B. We can now write the expected distance of a hybrid from the optimum, as

$$
\begin{aligned}
E\left(r^2_{\mathrm{HO}}\middle|\, h, p_{12}, \theta_E\right) =& r^2_{..} \\
& + \tfrac{1}{2}\theta_E\left(r^2_{.\mathrm{B}} - r^2_{.\mathrm{A}}\right) \\
& + \left(h - \tfrac{1}{2}\right)\left(r^2_{2.} - r^2_{1.}\right) \\
& + \tfrac{1}{2}\left(h - \tfrac{1}{2}\right)\theta_E\left(\left(r^2_{1\mathrm{A}} - r^2_{1\mathrm{B}}\right) - \left(r^2_{2\mathrm{A}} - r^2_{2\mathrm{B}}\right)\right) \\
& - p_{12} \\
& + h(1-h)\left(4 - r^2_{12}\right)
\end{aligned}
\tag{48}
$$

We can then take expectations over the indicator variable in eq. 48, so that $E(\theta_E) = 2q - 1$. In this way, we find

$$
\begin{aligned}
E_{PE}\left(r^2_{\mathrm{HO}}\middle|\, h, p_{12}\right) =& r^2_{.\mathrm{A}} + q\left(r^2_{.\mathrm{B}} - r^2_{.\mathrm{A}}\right) \\
& + \left(h - \tfrac{1}{2}\right)\left[r^2_{2\mathrm{A}} - r^2_{1\mathrm{A}} + q\left(r^2_{1\mathrm{A}} + r^2_{2\mathrm{B}} - r^2_{1\mathrm{B}} - r^2_{2\mathrm{A}}\right)\right] \\
& - p_{12} + 4h(1-h)\left(1 - \tfrac{1}{4}r^2_{12}\right)
\end{aligned}
\tag{49}
$$

where the subscript "*PE*" indicates a patchy ecotone. Equation 49 corresponds to results for a single environment (eqs. 9-14) when $q = 0$ or $q = 1$.

Now let us consider predictions for an environmental gradient. To model this, let us assume that the optimum experienced by the hybrids is intermediate between two other optima, A and B. Let us now treat $0 \leq q \leq 1$ as measure of the closeness of the relevant optimum to optimum B. With this definition, we can write the optimal value for trait i in the intermediate environment as

$$
o_i = o_{\mathrm{A},i} + q(o_{\mathrm{B},i} - o_{\mathrm{A},i}) \tag{50}
$$

where $o_{\mathrm{A},i}$ and $o_{\mathrm{B},i}$ are the optimal trait values in the two extreme habitats. Using this definition in eqs. 9-14, we find:

$$E_{EG}\left(r_{\mathrm{HO}}^{2}\middle|h,p_{12}\right)=E_{PE}\left(r_{\mathrm{HO}}^{2}\middle|h,p_{12}\right)-q(1-q)r_{\mathrm{AB}}^{2} \tag{51}$$

where the subscript "EG" denotes results at a point along an environmental gradient. Equations 49 and 51 differ only by the term $-q(1-q)r_{\mathrm{AB}}^{2}$. This implies that, with the quantitative genetics partition, all of the composite effects will be identical for these two sorts of environmental heterogeneity, if we use the appropriate definitions of q.

Appendix 2: Individual-based simulations

In this appendix, we present individual-based simulations of evolution and hybridization under Fisher's geometrical model. The aim is to support assertions in the main text, explore the robustness of our approximations, and explore the effects of the divergence process under an explicit population genetic model. The simulations reported also include those shown in main text Figures 4-5.

A2.1 The evolution of single populations

In the first part of the appendix, we describe the evolution of single populations under the additive version of Fisher's model (eq. 5). The aim is to clarify the meaning of some key quantities, especially $\|\mathbf{1}\|_{\lambda}^{2}$ and $r_{1\text{O}}^{2}$ (eqs. 7 and 8), and to show how they depend on population genetic parameters, and the history of environmental and demographic change.

A2.1.1 Basic simulation methods

We simulated the evolution of multi-locus diploid Wright-Fisher populations with selection. The population comprised N simultaneous hermaphrodites. Generations were discrete and non-overlapping. Each generation, $2N$ parents were chosen with replacement, with probabilities proportional to their fitness, as determined from eq. 3. Gametes were generated from each parental genome with recombination and mutation. Assumptions about recombination varied (see below), but the number of new mutations was drawn from a Poisson distribution, with mean $2NU$, and these were placed on the $2N$ gametes at random. The model assumed an effectively infinite number of sites, and so each new mutation took place at a unique, and randomly-chosen location in the genome, with no back mutation. Simulations began with an ancestral population that was genetically uniform, although standing variation could accumulate during the simulation. All simulation code was written in C, with random numbers generated using *MTRand* (Matsumoto and Nishimura, 1998), and is available as online supplementary information.

A2.1.2 Description and justification of population genetic parameter values

We simulated the model above in a range of parameter regimes. In particular, we varied six parameters, as described in Table A2.1. When the values were fully crossed, this yielded 128 distinct parameter

regimes.

The key population genetic parameters are the rates of recombination, $\bar{c}$ and mutation, U (Table A2.1). These determine the extent to which mutations will appear in a variety of genetic backgrounds during their fixation, and thus, the extent to which epistatic interactions might influence the divergence process. With this in mind, we compared very high and very low rates of recombination. In particular, we assumed either free recombination among all sites, or that all mutations were found on a single chromosome of map length 100cM with Haldane's mapping function. In other words, the number of crossover events was a Poisson random variable, with mean 1, and crossover locations were positioned at random. From Zeng (1990; see also eq. 9.3 of Lynch and Walsh 1998), this corresponds to a mean crossover fraction of

$$\bar{c} = \frac{1}{2} - \frac{2L - 1 + e^{-2L}}{4L^2} = \frac{1 - e^{-2}}{4} \approx 0.216... \tag{52}$$

where $L = 1$ is the map length in Morgans. This value compares to $\bar{c} = 0.5$ under free recombination, and is lower than all of the empirical estimates compiled and reported by Lynch and Walsh (1998) (see their Table 9.2). For mutation rates, we chose values over four orders of magnitude, such that, when $N = 1000$, $2NU$ was as high as 10, generating high levels of segregating variation, and as low at 0.01, such that mutations often reached fixation or loss, without appearing in a range of genetic backgrounds.

Other parameters affect the overall shape of the fitness landscape (Table A2.1). We assumed isotropic selection (i.e., no differences among traits), and set the overall strength of selection at $\alpha = \frac{1}{2}$ (eq. 3), but we varied the number of phenotypic traits, n, and the curvature of the fitness landscape, k (eq. 3). For the number of traits we chose to simulate under $n = 2$ and $n = 20$. As shown in a range of previous work, these values can lead to qualitatively different dynamics during divergence (e.g., Orr 2000; Poon and Otto 2000; Lourenço et al. 2011; Zhang 2012; Roze and Blanckaert 2014). In general, populations with $n = 20$ tend to have higher genetic loads, and a reduced ability to track moving environmental optima, while $n = 2$ maximizes the extent of stochastic correlations between trait values. The parameter k also has strong effects on the divergence (Roze and Blanckaert, 2014; Fraïsse et al., 2016). We chose the value $k = 2$, which leads to a Gaussian function. This is the most widely-studied version of the model, and has theoretical justification as a truncated Taylor expansion of an arbitrary function (Martin and Lenormand, 2006a; Martin, 2014), and some empirical support, from the distribution of new mutations (Martin and

Lenormand, 2006b; Martin et al., 2007; Manna et al., 2011). The value $k = 2$ is also analytically tractable, since log fitness is directly proportional to the squared Euclidean distance: $\ln w \propto \|\mathbf{z}-\mathbf{o}\|_\lambda^2$. We also chose $k = 6$, which yields a "table-like" fitness landscape, where small deviations from the optimum ($\|\mathbf{z}-\mathbf{o}\|_\lambda < 1$) all have similarly high fitness, while larger deviations ($\|\mathbf{z}-\mathbf{o}\|_\lambda > 1$), lead to a precipitous decline in fitness. This higher value of k is better able to account for the very strong negative epistasis observed in some experimental studies of speciation genetics (Fraïsse et al., 2016).

The final pair of parameters specify the distribution of mutational effects on the phenotype. In all cases, we assumed that mutations showed universal pleiotropy (Martin, 2014; Sella and Barton, 2019). For the homozygous effects, we used two approaches: a "top-down" approach (Poon and Otto, 2000), where mutational vectors took a random direction in the n-dimensional space, and with magnitudes drawn from some known distribution. We also used a "bottom-up" approach, where the mutational effect on each trait was drawn from an i.i.d. normal distribution. The bottom-up approach tends to suppress mutations of small effect unless n is very small (Orr, 2000; Wingreen et al., 2003). Arguably, this makes the approach biologically unrealistic with larger n (Orr, 2000), although it remains widely used. For both top-down and bottom-up approaches, we chose the remaining free parameter to fix the mean strength of selection against deleterious mutations appearing in an optimal genotype. We denote this quantity $\bar{s}$. If we denote the mutational effect on trait i as x_i, for the bottom-up approach, we assumed that x_i was normally distributed with a vanishing mean, and a variance equal to

$$\mathrm{Var}(x_i) = \frac{1}{2}\left(-\frac{\bar{s}}{\alpha}\frac{\Gamma(n/2)}{\Gamma((n+k)/2)}\right)^{2/k} \tag{53}$$

For the top-down approach, the magnitude of each mutation, $x \equiv \sqrt{\sum_{i=1}^n x_i^2}$, was randomly generated, such that $x^{k/2}$ was exponentially distributed. The mean value of the exponential distribution was then set at:

$$E\left(x^{k/2}\right) = \left(\frac{-\bar{s}}{\alpha k!}\right)^{1/k} \tag{54}$$

(Fraïsse et al., 2016). We varied the mean strength of selection, $\bar{s}$, choosing the values $\bar{s} = 10^{-2}$ and $\bar{s} = 10^{-4}$. This implied that, when $N = 1000$, we had either $N\bar{s} = -10$, or $N\bar{s} = -0.1$. As such, selection

was either effective on average, or ineffective on average. The procedures described above apply to the homozygous effects of new mutations. Under phenotypic additivity, the heterozygous effects on each trait were generated by halving these homozygous effects.

A2.1.3 Results with system drift

Let us first consider results with a stable population size of $N = 1000$, and a stationary phenotypic optimum. In this case, populations remain close to the phenotypic optimum at all times, but undergo genomic change, due to nearly neutral evolution and "system drift" (Rosas et al., 2010; Schiffman and Ralph, 2017). For each of the 128 combinations of parameter values (Table A2.1), we simulated two replicate populations, stopping the simulation when $d = 500$ mutations had reached fixation. Results are shown in Figure A2.1. The first two panels (Figure A2.1a-b), show the simulated values of two quantities that appear in our analytical results.

First, consider the scaling factor $\|\mathbf{1}\|_\lambda^2 = \sum_{i=1}^n \lambda_i$ (eq. 6; Figure A2.1a). By definition, the values of the λ_i parameters are set implicitly, because they depend on the size of the mutations that reach fixation during evolution (see main text, and Appendix 1 section A1.1). For our simulations, we estimated $\|\mathbf{1}\|_\lambda^2$ from the sample variances of the realized m_{ij} for each trait, then summed across traits: $\sum_{i=1}^n \mathrm{Var}_j\left(m_{ij}\right)$. This is not a general solution, but it works acceptably well for our choice of fitness function (i.e., independent selection of unit strength on all traits, which in the notation of Appendix 1 eq. 31 is A equal to the identity matrix). The results in Figure A2.1a show that $\|\mathbf{1}\|_\lambda^2$ was very highly correlated between the two replicates, and so its value was a reliable indicator of the set of population genetic parameters. Further examination showed that the rates of mutation and recombination (U and $\bar{c}$) have less influence on the value of $\|\mathbf{1}\|_\lambda^2$ than do the four remaining parameters: n, k, $\bar{s}$ and the choice of top-down or bottom-up mutations (Table A2.1). These four parameters are indicated in Figure A2.1 by color and shape (see also Figure A2.2a for the same quantity on a log scale). Overall, results suggest that the distribution of mutant fitness effects (which is influenced by all four parameters), is the major determinant of $\|\mathbf{1}\|_\lambda^2$.

Let us now consider the distance of the phenotype from the optimum: $r_{1\mathrm{O}}^2$ (eq. 8; Figure A2.1b). This quantity was measured from a genome carrying all and only the 500 fixed mutations. Results in Figure A2.1b show that $r_{1\mathrm{O}}^2$ is less replicable than $\|\mathbf{1}\|_\lambda^2$, due to the chance fixation of weakly deleterious mu-

tations. However, there are still clear differences between parameter regimes (see also Figure A2.2b for plots on a log scale). These differences partly reflect variation in $||\mathbf{1}||_\lambda^2$, which appears in the denominator in eq. 8, but also indicate intrinsic differences between parameter regimes in the ability of the population to stay close to the optimum.

The remaining panels in Figure A2.1 clarify the meaning of r_{1O}^2. Figure A2.1c plots all 2×128 simulated values of r_{1O}^2 against the corresponding measures of fitness, w (eq. 3). Results confirm that r_{1O}^2 is a scaled, and transformed measure of fitness. The simulated fitness values agree with previous studies of genetic load under Fisher's geometric model (Peck et al., 1997; Hartl and Taubes, 1998; Poon and Otto, 2000; Tenaillon et al., 2007; Lourenço et al., 2011; Roze and Blanckaert, 2014). However, they also confirm that similar levels of genetic load can be obtained with different distributions of fixed factors; i.e., identical values of w can correspond to different values of $||\mathbf{1}||_\lambda^2$ and r_{1O}^2. This implies that the outcome of hybridization cannot be predicted from fitness alone.

Figure A2.1d-e show the tendency of r_{1O}^2 to decline with the genomic divergence. Because d appears in the denominator of eq. 8, r_{1O}^2 decreases as d increases, unless phenotypic divergence also increases by a proportional amount. This is why r_{1O}^2 can be interpreted as the observed phenotypic divergence, divided by the amount of phenotypic divergence expected under a random walk with a comparable distribution of effect sizes (see main text Figure 3). The value of r_{1O}^2 helps to determine the extrinsic, environment-dependent effects on hybrid fitness (eq. 17). Its decline over time therefore explains the convergence to a pattern of intrinsic isolation (eq. 18).

A2.1.4 Results with demographic and environmental change

The results shown in Figure A2.1 assumed a stable population size and stable environmental conditions. In Figure A2.2, we relax those assumptions.

First, we repeated our simulations, but with a transient reduction in population size, such that population had a fixed size of $N = 100$ for the first $25/U$ generations, while all subsequent generations had $N = 1000$ offspring. The corresponding results are shown in the first row of plots (Figure A2.2a-d). Figure A2.2a shows that the bottleneck led to a slight increase in $||\mathbf{1}||_\lambda^2$ but only in some parameter regimes ($k = 2$ and $\bar{s} = 10^{-2}$; yellow points). Overall, the bottleneck had very little effect on the results.

Clearer differences were observed when we introduced a more extreme demographic change, reducing the population size to $N = 10$ for the entire simulation run. These results are shown in the second row of plots (Figure A2.2e-h). Figure A2.2e shows clear increases in $||\mathbf{1}||_\lambda^2$ for the runs with $\bar{s} = 10^{-2}$ (red and yellow points), reflecting the reduction in the efficacy of selection on a typical mutation (from $N\bar{s} = 10$ with $N = 1000$ to $N\bar{s} = 0.1$ with $N = 10$). As shown in Figure A2.2f, populations of size $N = 10$ also drifted further from the optimal phenotype. Most notably, with $k = 2$ and $\bar{s} = 10^{-4}$ (green points in Figure A2.2f), simulated values were close to the value of $r_{1O}^2 = 4$ (indicated by the dashed lines), which is the expectation under phenotypic neutrality. As shown by the green lines in Figures A2.2g-h, the value of $r_{1O}^2 \approx 4$ was maintained throughout the divergence process, indicating that stabilizing selection was ineffective, so that phenotypic evolution was indistinguishable from a random-walk. In other parameter regimes ($k = 6$ and/or $\bar{s} = 10^{-2}$), there was a decline in r_{1O}^2 with d, showing that stabilizing selection was not completely ineffective in these parameter regimes.

The third row of plots (Figure A2.2i-l) shows results when the population size was stable ($N = 1000$), but the phenotypic optimum shifted at the beginning of the simulation. To model this, the trait values of the initial population were all placed at the origin (such that all $z_i = 0$), but the optimum value of one of the n traits was set to 1. In consequence, the initial population had a fitness of $w = e^{-\alpha||\mathbf{z}-\mathbf{o}||_\lambda^k} = e^{-1/2} \approx 60\%$ relative to an optimal population. The effect was to induce an initial bout of adaptive substitution, as the population evolved towards its new optimum. Previous results suggested that this would increase the variation in effect sizes of the factors fixed (Orr, 1998b; Rockman, 2012; Matuszewski et al., 2014, 2015; Thompson et al., 2019; Yamaguchi and Otto, 2020). As shown in Figure A2.2i, an increase in $||\mathbf{1}||_\lambda^2$ is observed in some parameter regimes, although the effect was quite small. This is partly explained by the way that $||\mathbf{1}||_\lambda^2$ is defined, so that its value is not affected by any tendency for substitutions to point in a particular direction. In addition, the adaptation often required far fewer than 500 fixations, so that the adaptive substitutions formed a small proportion of the total. While $||\mathbf{1}||_\lambda^2$ is relatively unaffected by the bout of adaptive substitution, its effects are much more clearly evident in the values of r_{1O}^2, as shown in Figure A2.2j-l. First, in all parameter regimes, adaptation to the new optimum left the populations maladapted to the initial optimum. This is shown by the systematically higher values of r_{1O}^2 shown in Figure A2.2j. Second, the green lines in Figures A2.2k-l (with $k = 2$ and $\bar{s} = 10^{-4}$) show a clear tendency

for r^2_{1O} to increase in the initial stages of divergence. In these parameter regimes, adaptation to the new optimum took an appreciable number of substitutions, so that r^2_{1O} increased initially, before decreasing with the onset of system drift at the new optimum.

Finally, in the bottom row of plots (Figure A2.2m-p), we report simulations where we allowed the optimal value of one of the traits to change over time, in a smooth, cyclical fashion. To model this, we set the trait optimum at generation t to

$$o_i(t) = \frac{1}{2}\left[1 + \sin\left(\frac{2\pi t}{10^5} - \frac{\pi}{2}\right)\right] \tag{55}$$

This function oscillates over a period of 10^5 generations between 0 and 1. A consequence of the constantly moving optimum was that most substitutions became adaptively driven, and often in opposite phenotypic directions. As shown in Figure A2.2m, this led to appreciable increases in $||\mathbf{1}||^2_\lambda$ in most parameter regimes. In parameter regimes where the population was relatively slow to adapt, the value of r^2_{1O} also cycled over the course of divergence; this is shown in Figure A2.2o-p.

A2.2 Hybridization between divergent populations

The previous section shows how $||\mathbf{1}||^2_\lambda$ and r^2_{1O} reflect the population genetic parameters and the pattern of environmental change experienced by single populations. In this second section of appendix, we consider the divergence between pairs of populations, focussing on the phenotypic distance between them: r^2_{12} (eq. 15; main text Figure 3). We then simulate the formation of hybrids between the populations.

A2.2.1 Simulation methods for diverging population pairs

To simulate pairs of divergent populations in allopatry, we used the simulations described above, combining pairs of replicate runs. By relabelling the traits, or reversing the sign of phenotypic effects, we were able to consider populations experiencing different sorts of environmental change. Allopatric simulations were halted as soon as one of the two populations had fixed 500 substitutions, such that the total divergence was bounded at $500 \leq d \leq 1000$. The genotypes containing all and only fixed differences in each population were treated as the two parental lines: P1 and P2.

To explore the effects of parapatry, we also simulated evolutionary divergence with gene flow. To do

this, we followed the simulation procedure described above, but in each generation, with a probability m, each of the N offspring in a given population was formed from parents originating from the other population. We chose to simulate migration rates such that $Nm = 0.1$ and $Nm = 1$. Migration at these levels leads to different levels of differentiation under neutrality. We did not explore lower values, where dynamics tend to resemble allopatry, nor larger values, which tend to prevent genomic divergence, due to allele swamping (Griswold, 2006; Yeaman and Whitlock, 2011; Débarre et al., 2015; Dittmar et al., 2016).

Under parapatry, there are few if any fixed differences between the populations. However, there can be a build-up of highly-differentiated loci, due to the balance between migration, drift and local adaptation (Griswold, 2006; Yeaman and Whitlock, 2011; Débarre et al., 2015; Dittmar et al., 2016). Accordingly, we terminated parapatric simulations after $50/U$ generations, and then defined d as the number of alleles whose frequencies differed by >50% between the two populations. The parental lines were the genotypes containing the locally common alleles at all of these highly differentiated loci, plus any alleles that had fixed in both populations. In all cases, we confirmed that these parental genotypes were representative of the diverging populations, by comparing their phenotypes to the modal phenotype in their population.

A2.2.2 Results for phenotypic distance between the parental lines

We simulated divergence under 15 different scenarios of environmental and demographic change. These 15 scenarios are described below, and illustrated in Figures A2.3. Initial analysis showed that that 11/256 parapatric simulations led to very low levels of divergence ($d < 10$), and so these were excluded from further analysis. Results for the remaining $(128 \times 15) - 11 = 1,909$ pairs of populations are shown in figure A2.4.

First, let us consider divergence scenarios 1-4. As illustrated in Figure A2.3a, these scenarios involved local adaptation, with optima that moved in opposite phenotypic directions with respect to the initial optimum in the ancestral habitat. As shown in Figure A2.4a, the adaptive divergence in scenarios 1-4 led to some very high values of r_{12}^2. The r_{12}^2 values are highest when the divergence took place in the face of ongoing gene flow (scenarios 1-2), which prevented genomic divergence at loci that were not directly involved in adaptation to the new optima (Yeaman and Whitlock, 2011). This gene flow had

two consequences. First, the paths of fixed substitutions tended to point in the same direction, from the phenotypes of P1 to P2 (resembling panel (a) of main text Figure 3, more than panel (c)). Second, as shown in Figure A2.4b, gene flow kept the overall genomic divergence low. Both factors act to increase r_{12}^2. Nevertheless, the fixed mutations varied in size, so simulated values remained well below their theoretical maximum of $\max\left(r_{12}^2\right) = 4d$ (see Appendix 1 section A1.5). Figure A2.4a also shows that the r_{12}^2 values were higher when the optima moved in single jump (scenarios 1-3), than when it moved back and forth via smooth cycles (scenario 4). This reflects the meandering in the chains of substitutions when environmental conditions fluctuate (compare panels (a) and (d) in main text Figure 3).

A second feature of scenarios 1-4 is that the optimum in the ancestral habitat was exactly intermediate between the two new optima. As such, this habitat provided ideal conditions for hybrid advantage (Moore, 1977; Yamaguchi and Otto, 2020). This is illustrated in Figure A2.4d, where for all four scenarios, $r_{12}^2/(4r_{\cdot O}^2)$ was close to its maximal value of 1 (eq. 16). Deviations from this maximum were due to cycling optima returning to their initial positions (scenario 4), or to high levels of maladaptive gene flow moving the parental populations away from their local optima (scenario 1).

Let us now consider scenarios 5-6, which are illustrated in Figure A2.3b. In these cases, population P1 evolved, while population P2 remained in their common ancestral state. As shown in Figure A2.4a, the r_{12}^2 values for scenarios 5-6, closely resembled those for scenarios 3-4. This is because the phenotypic divergence was around half as large, but so was the genomic divergence (Figure A2.4b). As shown in Figure A2.4d, $r_{12}^2/(4r_{\cdot O}^2)$ was also reduced to exactly 1/2. This is because the position of the optimum in the ancestral habitat no longer maximized hybrid advantage: while hybrids would often be fitter than parental line P1, they could never be fitter than the optimal P2.

Very similar results were obtained for scenarios 7-8. In these cases, as illustrated in Figure A2.3c, populations adapted to moving optima on different traits. The values of $r_{12}^2/(4r_{\cdot O}^2) \approx 1/2$ (Figure A2.4d) reflected the fact that hybrids formed in the ancestral habitat could gain a fitness advantage over both parental lines, but less so than in a habitat whose optimum was intermediate between the parental phenotypes (see also main text Figure 4).

In scenarios 9-10, results were qualitatively different. Here, as illustrated in Figure A2.3d, the two populations adapted, independently, to identical environmental change. This scenario is often associated

with "mutation-order speciation" (Mani and Clarke, 1990; Gavrilets, 2004). As a result, the two parental phenotypes were very similar to each other (such that $r_{12}^2 \ll 1$).

The next group of scenarios, 11-13, assumed stable environmental conditions, so that all of the divergence accumulated via genetic drift in allopatry. When population size was consistently small (scenario 11), populations were able to wander away from their optima via genetic drift (Figure A2.3e). In some parameter regimes, this led to high values of r_{12}^2 (Figure A2.3a), consistent with the single-population results (Figure A2.2e-h). By contrast, when population sizes were higher (with or without a transient bottleneck), diverging populations remained close to their phenotypic optima (Figure A2.3f), and so r_{12}^2 remained low (Figure A2.4a). In all three scenarios, the values of $r_{12}^2/(4r_{.\text{O}}^2)$ varied greatly (Figure A2.4d). This is because, under drift, parental populations deviate from the optimum in independent, random directions.

Finally, for scenarios 14-15, we re-analysed the simulations from scenarios 3-4, but scored hybrids in a "benign environment" (Hatfield and Schluter, 1999). This means that fitness was affected only by the $n-1$ phenotypic traits that had not been subject to diversifying selection. As such, the fitness of hybrids was determined by the pleiotropic effects of adaptive substitutions (Thompson, 2019), and results closely resemble those under "system drift".

Taken together, the single-population results (Figures A2.1-A2.2) and the two-population results (Figures A2.3-A2.4) paint a consistent picture. The scaling factor, $||\mathbf{1}||_{\lambda}^2$, is mainly determined by the population genetic parameter regime, while the scaled phenotypic distances, such as r_{12}^2, are affected in predictable ways by both the parameter regime, and the demographic and selective history of the diverging populations.

High values of r_{12}^2, which are conducive to hybrid advantage, can be generated by genetic drift, but only when phenotypic selection is ineffective. High values can also be generated by natural selection, but only when the environment changes in certain ways. The very highest values occur under adaptive substitution in opposite phenotypic directions, and especially when divergence took place in the face of gene flow.

A2.2.3 The robustness of the Brownian bridge approximation

To explain patterns in hybrid fitness, we have used a Brownian bridge approximation (eqs. 9-14). Under this approximation, all of the differences between divergence scenarios, and population genetic parameter regimes, should captured by the quantity, M, which depends on the phenotypic distances $r^2_{1\mathrm{O}}$, $r^2_{2\mathrm{O}}$ and r^2_{12} (eq. 14). By contrast, the quantity V, which captures the variability in the effects of P2 alleles, is predicted to be identical under all scenarios (eq. 12). For homozygous hybrids (where $p_{12} = 0$), its value should be $V = 4h(1-h)$, and so depend solely on the hybrid index h (eq. 12).

We tested this prediction using the 1,909 pairs of parental lines shown in Figure A2.4. We generated 10,000 homozygous hybrids, for each pair of lines, and each of 25 different values of h. In each of these hybrid genomes, we added hd P2 alleles to an otherwise P1 background. The alleles were chosen at random without replacement from the d divergent loci, and were added in homozygous state. Next, for each pair of lines, and each value of h, we used the simulated values of $r^2_{1\mathrm{O}}$, $r^2_{2\mathrm{O}}$ and r^2_{12} to calculate the quantity M (eq. 14), and then estimated V (eq. 10) from $\hat{V} = \overline{r^2_{\mathrm{HO}}} - M$ (where the overbar denotes the sample mean across the 10,000 hybrids). Finally, we compared these estimates to the prediction from the Brownian bridge approximation. Results are shown in Figure A2.5. Figure A2.5 shows that the simulated values agree well with the prediction for all divergence scenarios, and all sets of population genetic parameters. The only visible deviations are for the subset of parapatric runs where there was low genomic divergence (i.e., $d < 50$; see Figure A2.4b). Agreement with the predictions was excellent for all runs where $d > 100$.

The derivation of the Brownian bridge assumed multivariate normality among the fixed effects (see Appendix 1 sections A1.1-A1.3). However, central limit type effects imply that the approximation should work well even when there are deviations from multivariate normality. To demonstrate this, we examined the scaled standard deviation in r^2_{HO}. In this case, we also have an analytical prediction of $4h(1-h)$ (see Appendix 1, eq. 43 for details), but this prediction relies much more strongly on the assumption of normality. Figure A2.6 plots the simulation results for the scaled deviation in r^2_{HO}. Figure A2.6 shows that the simulations match the predictions only for scenarios 12-15 (system drift and pleiotropic effects; Figure A2.6l), suggesting that fixed effects were close to multivariate normal in these simulations. For all other scenarios (Figure A2.6a-k), agreement with the prediction was often poor. As expected from

previous work (Orr, 1998b; Rockman, 2012; Matuszewski et al., 2014, 2015; Thompson et al., 2019; Yamaguchi and Otto, 2020), agreement was worst when substitutions took place by positive selection (scenarios 1-10; Figure A2.6a-j), and especially with a transient burst of adaptive change (scenarios 1-3, 5, 7 and 9). The strong contrast between Figures A2.5 and A2.6 shows that the basic Brownian bridge approximation works well even when there are strong deviations from multivariate normality among the fixed effects (Simon et al., 2018).

A2.2.4 Results for lines crosses

The purpose of the Brownian bridge approximation is to make predictions for particular classes of hybrid genotype, at different levels of divergence, and under different environmental conditions. This is illustrated in Figure 4 of the main text. The simulations in Figure 4 used divergence scenario 7 (Figures A2.3c and A2.4); in other words, the populations diverged in allopatry, by adapting towards distinct and distant phenotypic optima, affecting the values of different phenotypic traits. Figures A2.7 and A2.8 show comparable results for some of the other divergence scenarios. In Figure A2.7, populations diverged under scenario 9 (Figure A2.3d, and Figure A2.4). This means that they adapted, independently, to the same environmental change (as in the classic scenarios of mutation-order speciation; Mani and Clarke 1990; Gavrilets 2004). They key fact about scenario 9 is that it yields $r_{12}^2 \approx 0$ (Figure A2.4a). As such hybrids always showed a pattern of intrinsic isolation equivalent to eq. 18. The sole effect of varying environmental conditions is to offset the results according to $r_{.\mathrm{O}}^2$: the parental maladaptation in the current environment.

Figure A2.8 shows results with divergence under drift, corresponding to scenarios 11-12 (Figure A2.3e-f). With effective stabilizing selection, and system drift (Figure A2.8a), there is rapid convergence to the characteristic pattern of intrinsic isolation (eq. 18). When stabilizing selection is ineffective (Figure A2.8b), most genomic divergence comprises deleterious mutations. In this case, there is also a lot of stochasticity, but we have $E\left(r_{.\mathrm{O}}^2\right) = 2$, and $E\left(r_{12}^2\right) = 4$, such that $\{\alpha_2\} \approx 0$. In these conditions, there is no effect of admixture *per se*, because P1 and P2 alleles are equally likely to be deleterious, and have no tendency to interact favorably with their original genetic background. However, the deleterious mutations are recessive on average (Wright, 1922, 1929; Manna et al., 2011), so that hybrid fitness increases with

heterozygosity. Results in this parameter regime agree with predictions from the classical theory of inbreeding (Wright, 1922; Simon et al., 2018).

All of the hybrids shown in Figures 4, A2.7 and A2.8 were generated under the assumption of free recombination in the F1 gametes. However, the predictions of eq. 17 are generally robust to reducing the rate of recombination. To show this, Figure A2.9 repeats the results of main text Figure 4, but with divergence and hybridization both simulated under limited recombination ($\bar{c} \approx 0.219$; eq. 52). Results show that eq. 17 remains a good approximation, as long as $\mathrm{Var}(h)$ is correctly calculated.

A2.3 Variable phenotypic dominance

In this third and final section of the appendix, we present simulations incorporating variable phenotypic dominance. The aim is to test the approximations of eqs. 26-28, and to demonstrate the key differences in the patterns of hybrid fitness.

A2.3.1 Simulations with variable phenotypic dominance

We repeated our complete set of 15×128 simulations as described above, except for one difference. The heterozygous effects of each new mutation were determined by multiplying its n homozygous effects (generated as described above) by n uniformly-distributed random numbers. As a results, the average levels of dominance of new mutations were unchanged, at $1/2$, but, unlike in the additive model, there was variation around this value.

We note that these uniformly-distributed random numbers do not correspond directly to the δ_{ij} values in eq. 25. This is because the δ_{ij} (like the m_{ij}), refer to fixed effects, and not to new mutations. As such, their distribution is determined by the process of evolutionary divergence.

A2.3.2 Variable phenotypic dominance and the Brownian bridge approximation

Variable phenotypic dominance is predicted to affect both terms in the Brownian bridge approximation (eqs. 26-28), although the changes should only affect hybrids with heterozygous loci.

To see this, Figure A2.10 shows results for the terms which capture variation in the P2 alleles (eqs. 12 and 27). As described above (section A2.2.3), results were generated by adding randomly-chosen

P2 alleles into a P1 background. But Figure A2.10 compares results when alleles were added in homozygous state (panels (a)-(b)), or in heterozygous state (panels (c)-(d)); and also compares the additive model (panels (a) and (c)), to a model with variable phenotypic dominance (panels (b) and (d)). Results for homozygous hybrids are shown in Figure A2.10a-b. In this case, phenotypic dominance makes no difference: the basic Brownian bridge approximation gives good, and near identical results for both sets of simulations.

Results for heterozygous hybrids are shown in Figure A2.10c-d. With phenotypic additivity (Figure A2.10c) the heterozygous effects are exactly half of the homozygous effects, and so the prediction $V = p_{12}(1-p_{12})$ works well when $p_2 = 0$ (see eq. 11). With variable dominance (Figure A2.10d), results are very different. In this case, variability in the dominance effects, the δ_{ij}, add a new source of variance, as captured by the parameter v (eq. 27).

Because these results in Figure A2.10d differ markedly between divergence scenarios, they are plotted separately in Figure A2.11. The solid black lines in Figure A2.11 show predictions from eq. 27 after setting $v = 1/12$. This value matches the variance of the uniform distribution that was used to generate the dominance effects of new mutations. It is notable that this value gives reasonable results for some scenarios, and for some runs under most scenarios. However, there is also clear variation, showing that the selective sieve can act on the dominance effects. Furthermore, in a handful of simulations the variability is less than the predictions of the additive model (shown as black dotted lines), naively implying $v < 0$. It is notable these simulations runs all involved abruptly shifting optima (i.e., scenarios 1-3, 7 and 9), and/or unbalanced rates of evolution between the populations (scenarios 5-6). In these runs, one (but not both) of the populations fixed several large-effect dominant mutations. This led to strong directional dominance, and low variance in the heterozygous effects of the P2 alleles. As such, these results imply covariance between homozygous and dominance effects, and this was not included in our analytical treatment.

Figures A2.10 and A2.11 show the effects of variable phenotypic dominance on the first terms of the Brownian bridge approximation (V and V_δ; eqs. 12 and 27). Its effects on the remaining terms (M and M_δ: eqs. 14 and 28) have a greater biological significance. The most important effect is illustrated in Figure A2.12. Figure A2.12 shows the terms M and M_δ changing over a large number of generations. Figures A2.12a-b, show that M tends to vanish over time under the additive model. This is due to the

decline of the distances r^2_{1O}, r^2_{2O} and r^2_{12} as d increases (see Figures A2.1, A2.2 above, and main text Figures 3 and 4). Because M does not depend on the heterozygosity (p_{12}), the effect is identical for homozygous hybrids (Figure A2.12a) and for heterozygous hybrids (Figure A2.12b).

With variable phenotypic dominance things are qualitatively different. For homozygous hybrids, a decline towards zero is still observed (Figure A2.12c). However, for heterozygous hybrids, there is a plateau at a non-negligible value, even when divergence is very large (Figure A2.12d). As discussed in the main text, the reason for this difference is that, with phenotypic additivity, the average balanced hybrid will have the midparental phenotype. The distance from the optimum of this phenotype is given by $r^2_{PO} = r^2_{.O} - \frac{1}{4}r^2_{12}$, and so its value will decline along with the parental distances. With variable phenotypic dominance, a heterozygous balanced hybrid will manifest the complete set of dominance effects (the δ_{ij}). Because this globally heterozygous genotype is never subject to selection, its phenotype can wander steadily away from the optimum as divergence accrues. This implies that its distance from the optimum, r^2_{GO} has no tendency to decline with divergence. Figure A2.13 compares the values of r^2_{PO} and r^2_{GO} for our complete set of simulations with variable dominance. While there is some stochasticity, as expected, there is a clear tendency for $r^2_{GO} > r^2_{PO}$ to hold, regardless of the divergence scenario, or the population genetic parameter regime.

A2.3.3 Results for line crosses with variable phenotypic dominance

As discussed in the main text, variable phenotypic dominance introduces three qualitative differences to patterns of hybrid fitness. These differences are evident in simulated crosses. Figure A2.14 contains a set of simulated crosses, identical to those shown in Figure 4, except that the divergence and hybridization were simulated under the assumption of variable phenotypic dominance. Comparison of Figure 4 to Figure A2.14 shows all three of the differences. First, in Figure A2.14, the initial F1 cross (which is equal to the global heterozygote) has reduced fitness at high levels of divergence (compare, for example, A2.14i to Figure 4n). Second, unlike in the additive model, the effects of heterozygosity are now environment dependent (extrinsic), and so may change with the position of the optimum. This is clear from comparing the middle crosses (F1/F2/Fn) in Figure A2.14a and A2.14b. In the environment to which P1 is adapted, the F1 and F2 have similar fitness (Figure A2.14a), but the same crosses have different fitnesses in the

environment to which P2 is adapted (Figure A2.14b). (An equivalent difference is evident betweens panels (d) and (e)). Third, the hybrids in Figure A2.14 show “directional dominance”, such that P2 alleles are slightly more recessive or dominant, on average. Directional dominance implies that the reciprocal backcrosses to the two parental lines, may have different fitnesses, even when the parental lines themselves have identical fitnesses. This effect is evident, albeit weakly, in Figure A2.14c-e.

Table A2.1: Population genetic parameter values used in the simulations

Parameter	#Conditions	Description
**1. Population genetic parameters:**		
Recombination rate	2	Free recombination ($\bar{c} = 0.5$), or a single chromosome of map length 100 cM ($\bar{c} = 0.216..$)
Mutation rate, U	4	Values chosen so that $2NU = 10, 1, 0.1$ or 0.01 when $N = 1000$
**2. Fitness landscape parameters:**		
Curvature, k	2	$k = 2$ or 6 (quadratic or "table-like"; see eq. 3)
Number of "traits", n	2	$n = 2$ or 20
**3. Distribution of new mutations:**		
Homozygous effects:	2	Either "bottom-up" (i.i.d. normal on each trait), or "top-down" (random orientations and leptokurtic magnitudes)
	2	Variance set such that $\bar{s} = 10^{-2}$, or 10^{-4}, in an optimal phenotype (effective/ineffective selection with $N = 1000$)

Appendix 2 Figures

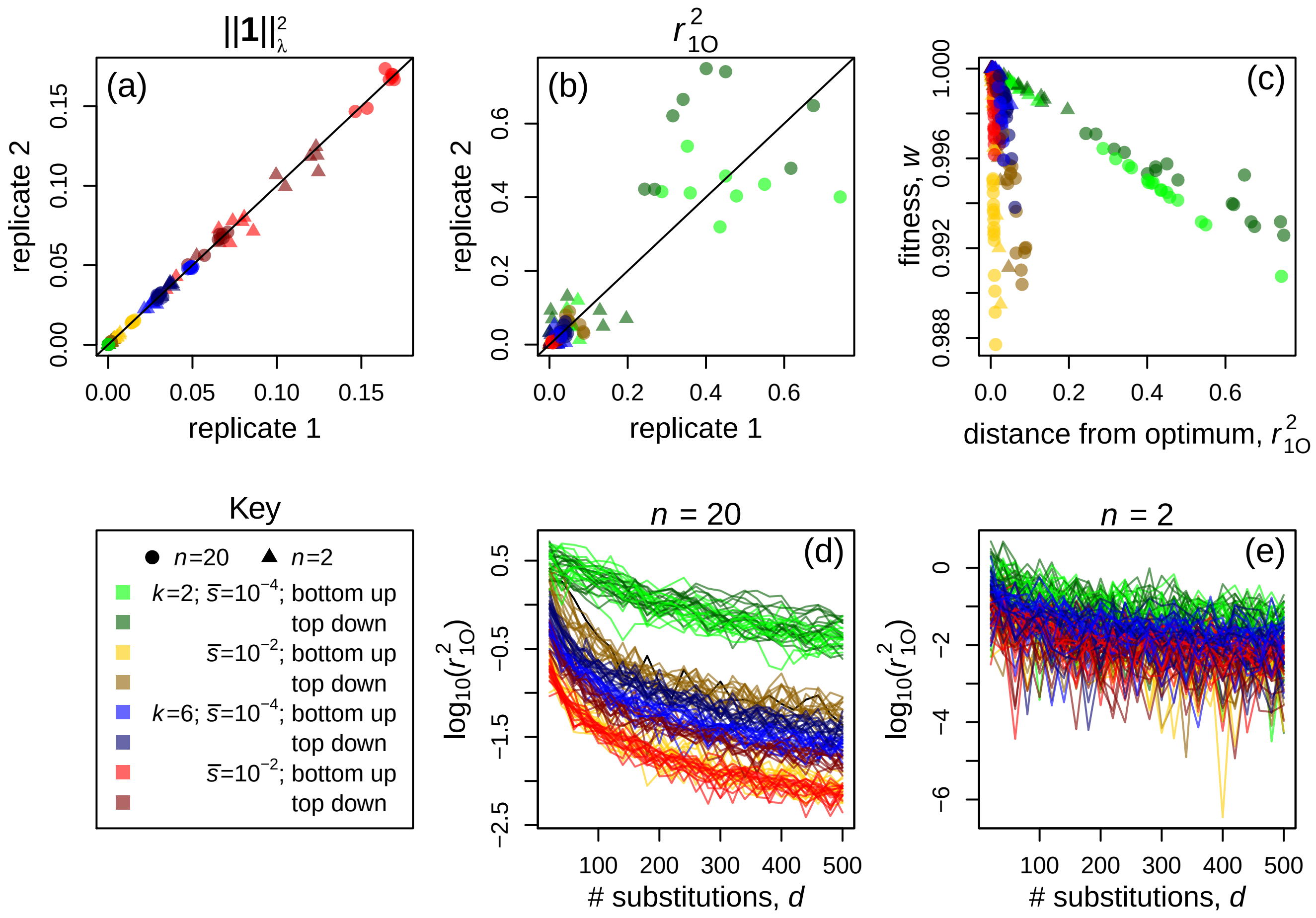

Figure A2.1 Simulated evolution of a single population with a stationary phenotypic optimum, and a stable population size of $N = 1000$. This yields effective stabilizing selection on the phenotype, but ongoing genomic evolution via "system drift". Each point shows results with 1/128 distinct sets of population genetic parameters (see Table A2.1), and the values of the four most influential parameters are indicated by color and shape. All simulations were stopped as soon as $d = 500$ mutations had reached fixation. **(a)** Simulated values of the scaling factor $\|\mathbf{1}\|_\lambda^2$ (eq. 7), as estimated from the variances in size of the 500 fixed effects (see text). **(b)** the distance of the final phenotype from the optimum, r_{1O}^2, where "distance" is defined according to eq. 8. **(c)** The relation between the phenotypic distance, and genotypic fitness, w (eq. 3). **(d)**-**(e)** The distance r_{1O}^2 tends to decline over time, indicating that the genomic divergence was not matched by proportional levels of phenotypic divergence.

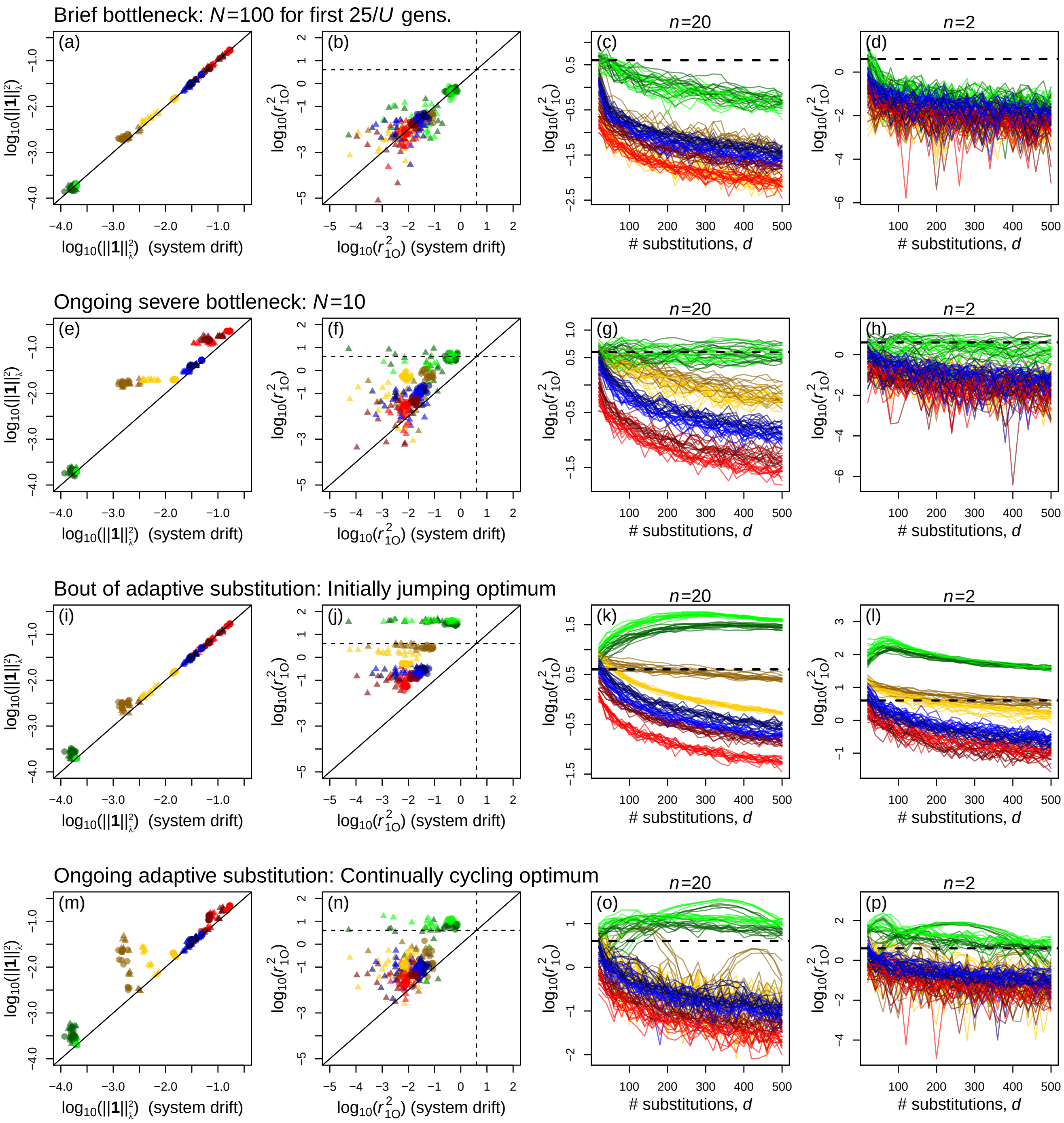

Figure A2.2 Simulated evolution of single populations under demographic and environmental change. Each row of four plots corresponds to a different sort of change, as described in the text. The first two columns compare simulation results to the equivalent results under system drift, as shown in Figure A2.1. All other details match Figure A2.1.

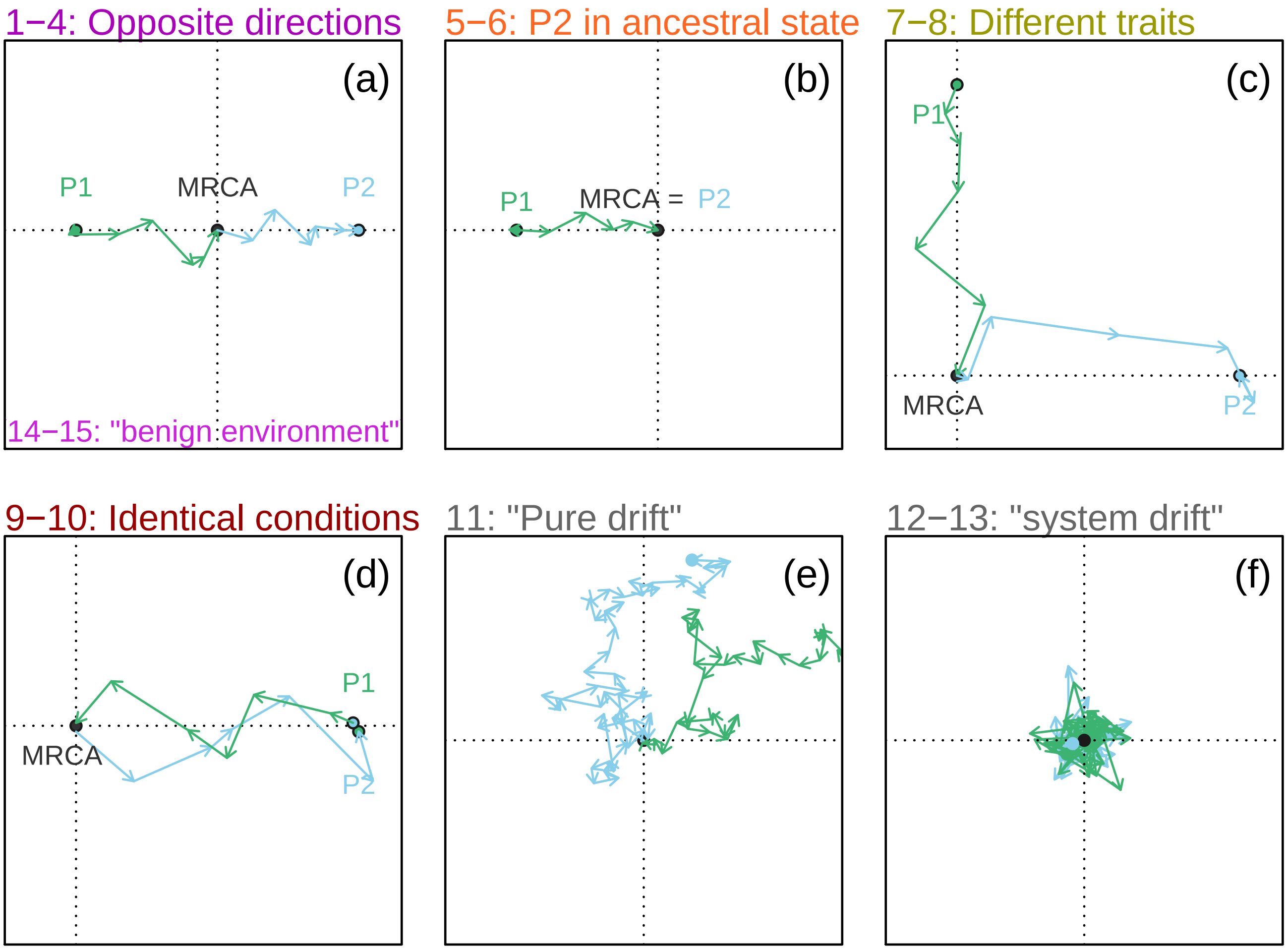

Figure A2.3 Illustrations of the 15 divergence scenarios that were simulated. For visual clarity, each scenario is illustrated with $n = 2$ traits, and low levels of genomic divergence, d. (Further details of the scenarios are given in the text, and Figure A2.4). **(a)** Scenarios 1-4: the two populations evolved towards two different optima, which differed from the ancestral phenotype by equal amounts, but in opposite phenotypic directions. (Scenarios 14-15 used the data from scenarios 3-4, but hybrid fitness was scored in a "benign environment", where fitness depended only on the $n-1$ phenotypic traits that were not subject to divergent selection pressures). **(b)** Scenarios 5-6: one population adapted to a distant optimum, while the other remained in their common ancestral state. **(c)** Scenarios 7-8: the two populations evolved towards different optima, involving different phenotypic traits. This meant that the ancestral phenotype was no longer intermediate between the two new optima. **(d)** Scenarios 9-10: the two populations adapted to shifting optima that moved identically, such that genomic divergence was due to the fixation of different sets of mutations, each conferring adaptation to the same environmental change. **(e)** Scenario 11: The optima remained stationary and matched the ancestral phenotype, but population size crashed to $N = 10$ individuals, such that stabilizing selection was often ineffective, and populations wandered away from the optimum. **(f)** Scenarios 12-13: The optima remained stationary, matching the ancestral phenotype; population sizes were sufficiently large for stabilizing selection to be effective.

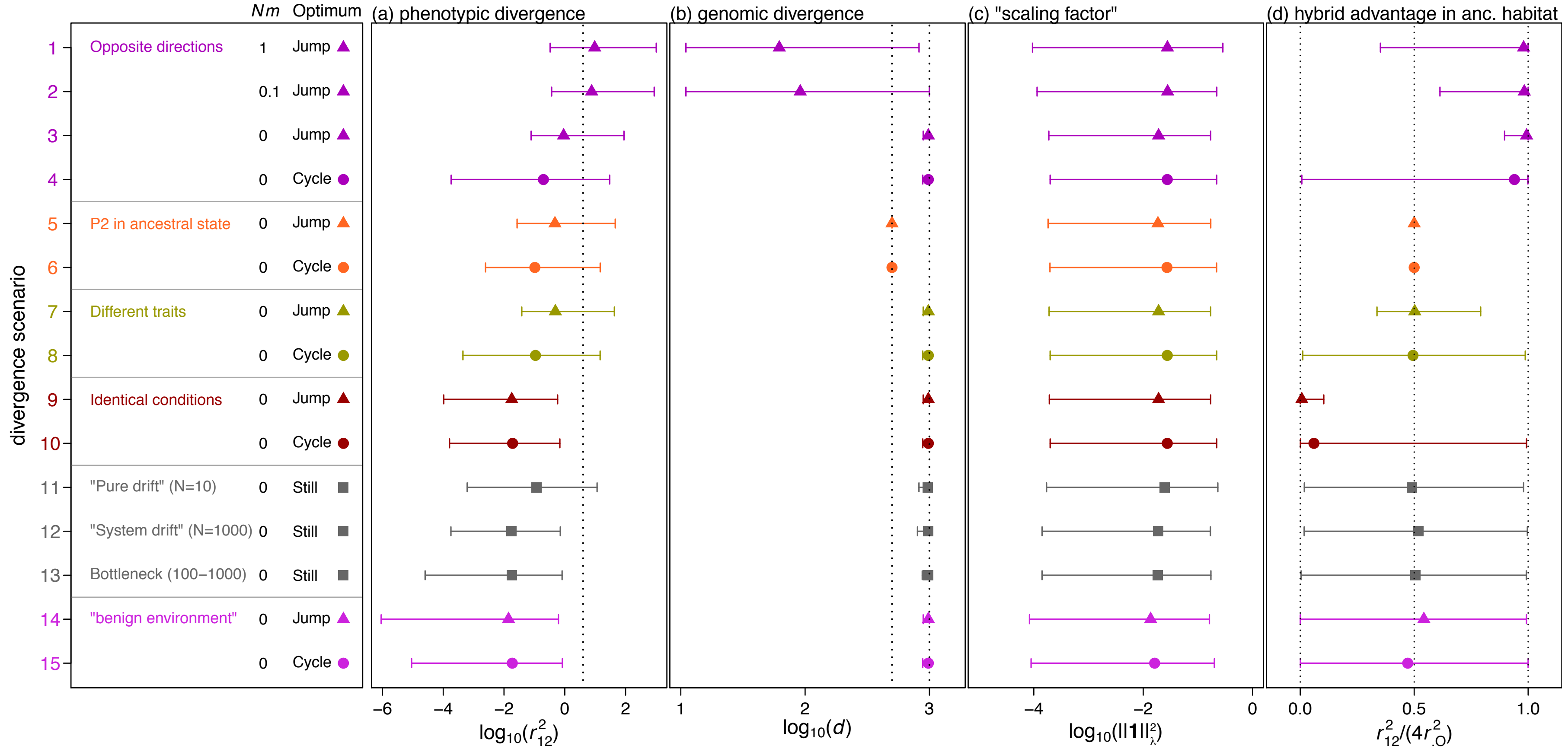

Figure A2.4 Simulated divergence between pairs of populations. Simulations used 15 scenarios of demographic and environmental change. These scenarios differed in the rate of gene flow during divergence (parameterized by the expected number of migrants, Nm), and in the environmental conditions. In particular, optima moved in a single abrupt jump (triangular points), in smooth repeating cycles (circular points), or remained stationary at the position of the ancestral phenotype (square points). Patterns of environmental and demographic change also varied, as indicated by color, and illustrated in Figure A2.3. Columns **(a)**-**(d)** show four quantities that determine the outcome of hybridization, with points and intervals showing the median and complete range of 128 simulations runs, each with a different set of population genetic parameters (see Table A2.1). **(a)** the log transformed distance between the parental phenotypes, r^2_{12}; the vertical dashed line shows the value $r^2_{12} = 4$, above which admixture confers a net advantage (eq. 17). **(b)** the genomic divergence, d; this quantity varied among the parametric runs (scenarios 1-2), according to the number of highly differentiated loci that were maintained by selection-migration-drift balance. Allopatric runs (scenarios 3-15) were terminated as soon as either population had fixed 500 substitutions. **(c)** the log transformed scaling factor, $\|\mathbf{1}\|^2_\lambda$, whose value was little affected by the divergence scenario. **(d)** the ratio $r^2_{12}/(4r^2_{.O})$ measured in the ancestral habitat (whose optimum matches the ancestral phenotype). This quantity is maximized at 1, when the position of ancestral optimum matches the midparental phenotype, thereby maximizing the fitness advantage for balanced hybrids.

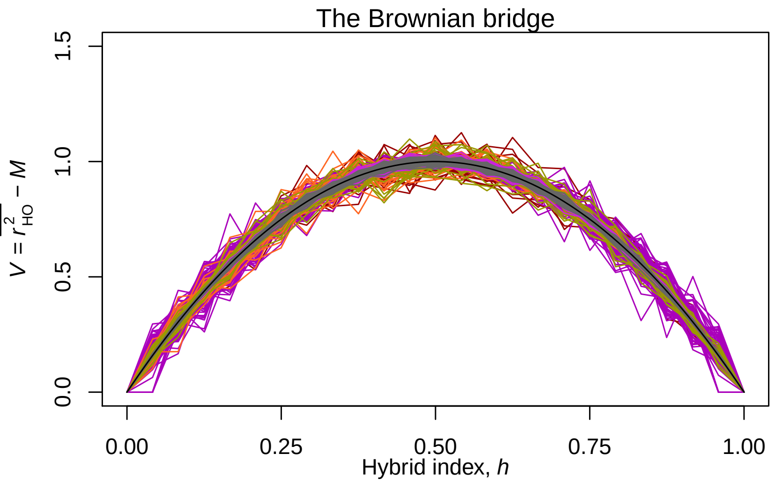

Figure A2.5 The robustness of the Brownian bridge approximation. Results show the first term of the approximation, V, which is unaffected by the conditions of divergence. The black line shows the analytical prediction of $V = 4h(1-h)$, which applies to homozygous hybrids (with $p_{12} = 0$). This prediction is compared to hybrid genomes generated from each of the 1,909 pairs of simulated populations, also used in Figure A2.4. Each pair of populations diverged under a different combination of population genetic parameters and environmental change (line colors correspond to the divergence scenarios described in Figures S2.3 and S2.4). The agreement with predictions is always good, and especially when genomic divergence was reasonably high ($d > 100$).

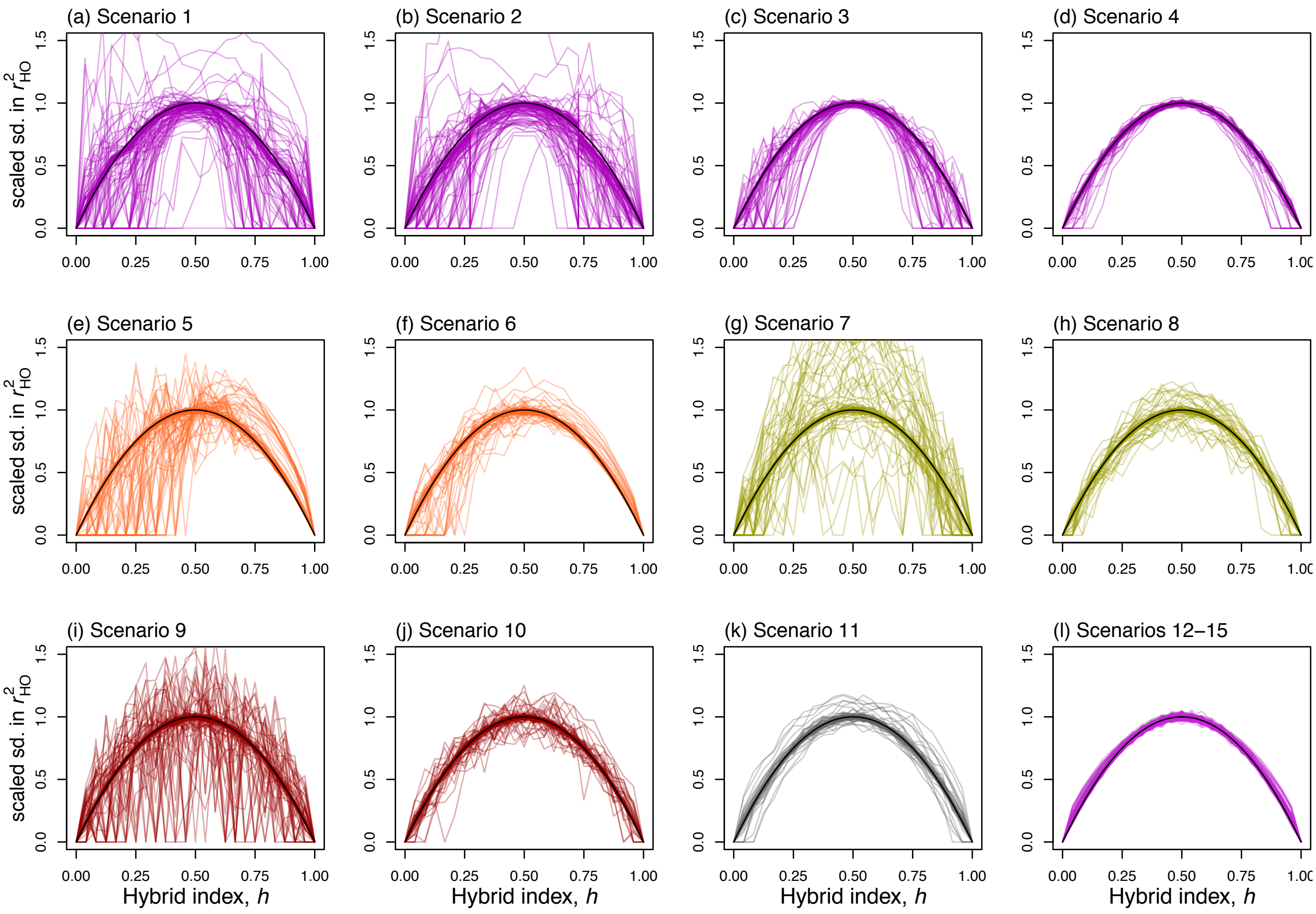

Figure A2.6 Evidence for deviations from multivariate normality in the fixed effects, that differentiate the simulated populations. All plots shows the scaled standard deviation in the distance from the optimum, r^2_{HO}. As in Figure A2.5, this was estimated from 10,000 homozygous hybrids, and various values of the hybrid index, h. Simulated values are compared to the analytical prediction of eq. 43, which depends strongly on the assumption of multivariate normality. Each panel contains results from one or more of the divergence scenarios, as described in Figures A2.3-A2.4. Agreement with predictions is poor for scenarios 1-10, suggesting that adaptive evolution leads to deviations from normality among the fixed effects.

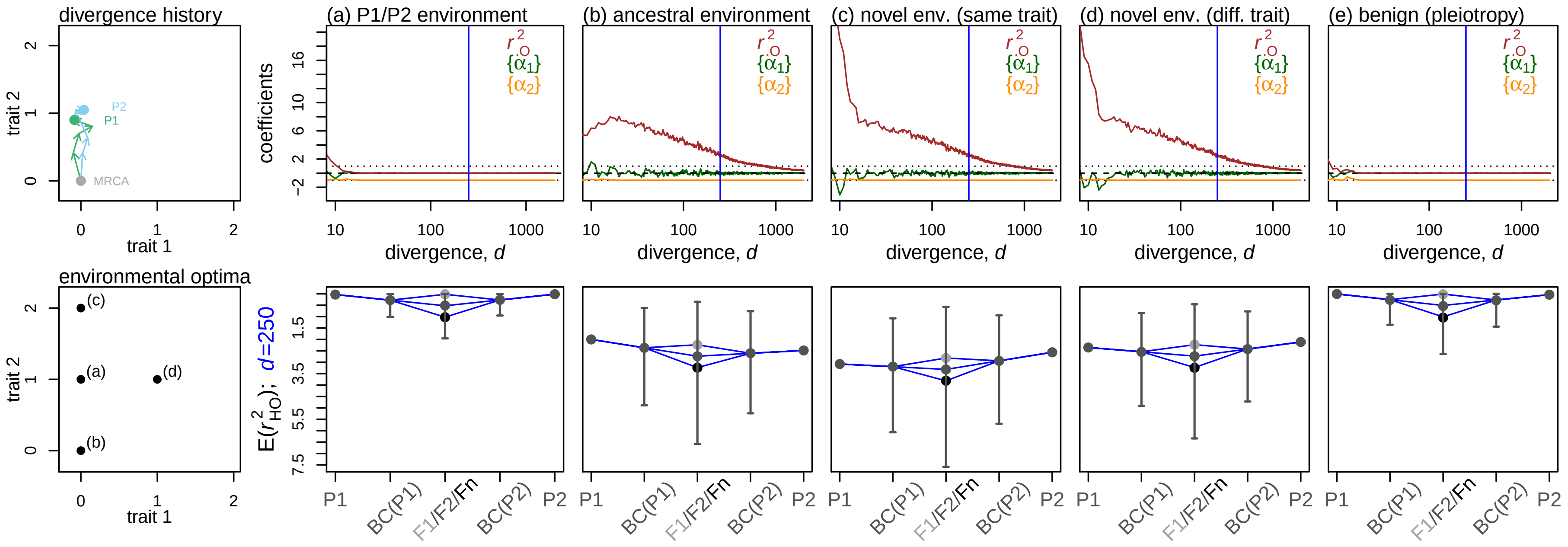

Figure A2.7 Simulated crosses in different environments. The parental populations adapted, independently, to identically shifting optima (this is shown, in cartoon, in the upper left-hand panel, and corresponds to "scenario 9" of Figure A2.4). **(a)-(d)** results for hybrids scored in four distinct environments, as indicated in the lower left-hand panel. **(e)** results in a "benign" environment, where the trait with the shifting optimum no longer affected fitness (as in scenarios 14-15 in Figure A2.4), so that hybrid fitness depended solely on pleiotropic effects on the remaining trait. All other details match main text Figure 4.

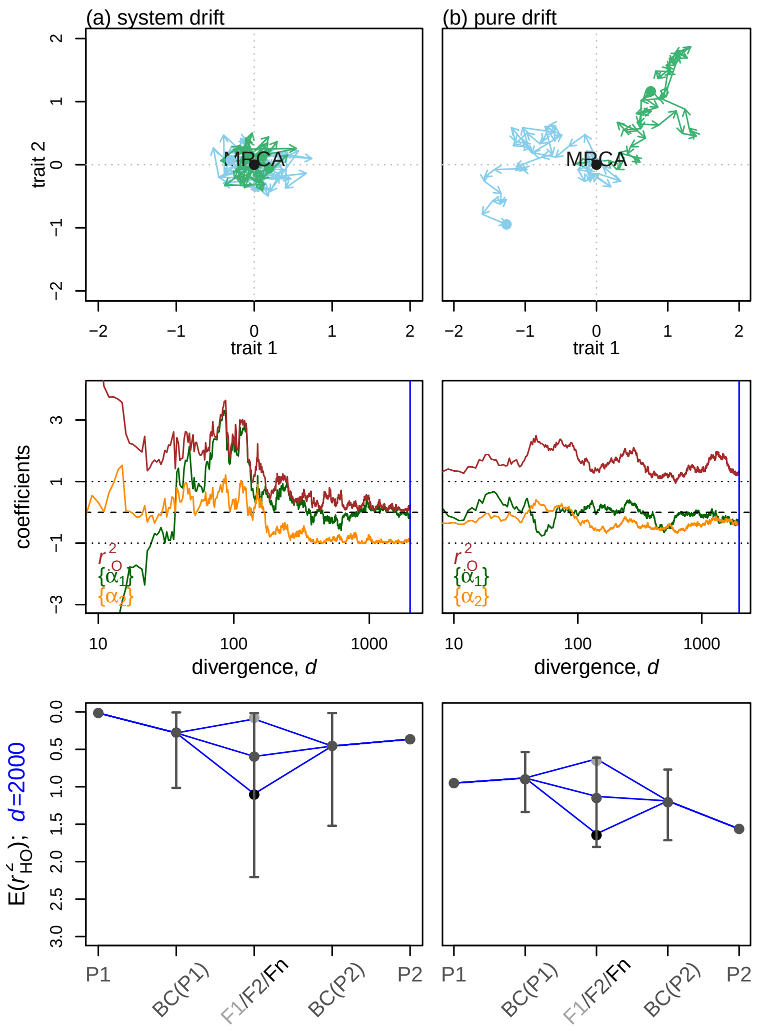

Figure A2.8 Simulated crosses when parental populations diverged via drift, using divergence scenarios 11-12 from Figure A2.4. **(a)** with "system drift", the populations remained close to the phenotypic optimum, but underwent nearly-neutral genome evolution. **(b)** with "pure drift", selection was so ineffective that populations continued to accrue mutations regardless of their effects. Population genetic parameters match those used in Figures 4 and A2.7, with the following exceptions **(a)**: $N = 100$, $N\bar{s} = -0.01$ and $n = 2$; **(b)**: $N = 50$, $N\bar{s} = -0.005$ and $n = 20$. All other details match main text Figure 4.

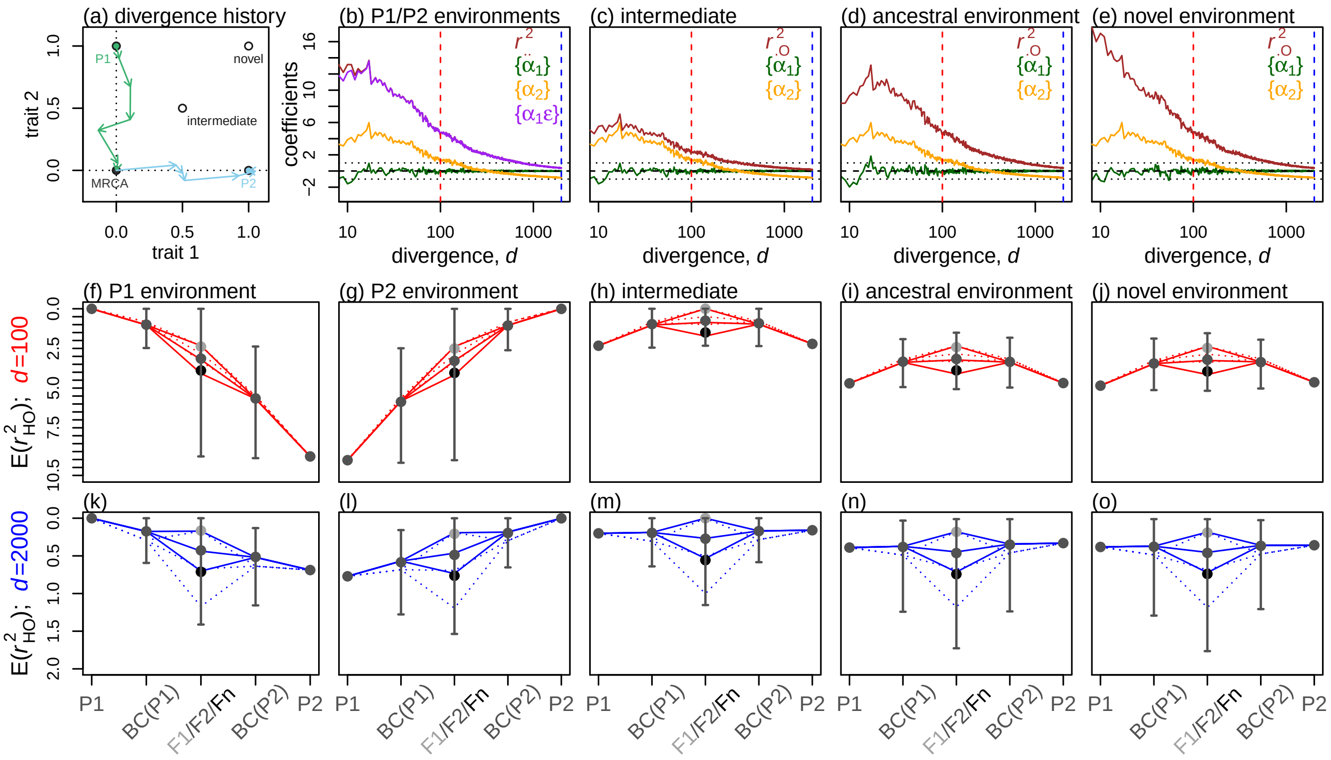

Figure A2.9 Simulated crosses after local adaptation, in populations with low recombination. All details match main text Figure 4, except for the levels of recombination assumed during the simulations. While Figure 4 assumed free recombination among all loci ($\bar{c} = 0.5$), here, during both divergence and hybridization, we assumed a single chromosome of map length 100cM and Haldane's mapping function. This corresponds to an average recombination rate of $\bar{c} \approx 0.216$ between randomly chose pairs of sites (see eq. 52). Analytical predictions used main text eq. 17 with $\text{Var}(h) = (1 - 2\bar{c})/4$ for the homozygous "Fn" (produced by amphimictic selfing among F1 gametes), $\text{Var}(h) = (1 - 2\bar{c})/8$ for the F2, and $\text{Var}(h) = (1 - 2\bar{c})/16$ for the backcrosses. For comparison, analytical predictions with free recombination are shown as dotted lines.

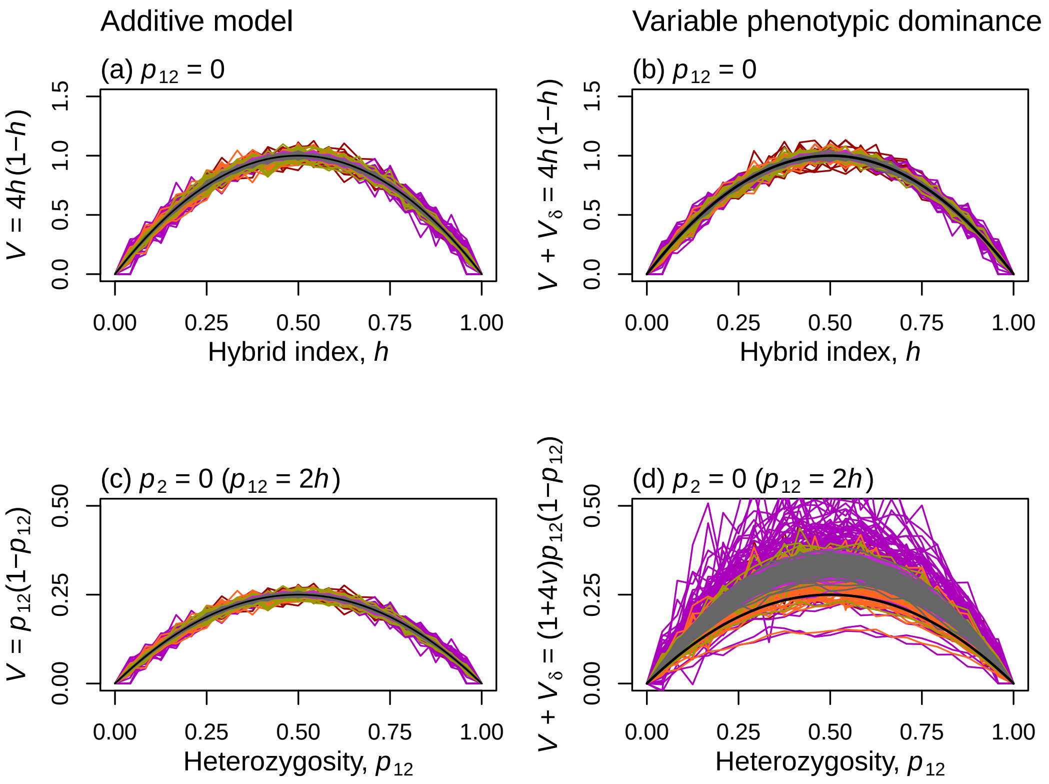

Figure A2.10 The Brownian bridge approximation with and without variable phenotypic dominance. **(a)** results with an additive phenotypic model, and homozygous hybrids (eq. 12 with $p_{12} = 0$), thereby replicating Figure A2.5. **(b)** results with variable phenotypic dominance and homozygous hybrids; these match the additive results because, from eq. 27, $V_\delta = 0$ when $p_{12} = 0$. **(c)** results with an additive phenotypic model, and heterozygous hybrids (eq. 11 with $p_2 = 0$, such that $V = p_{12}(1 - p_{12})$). **(d)** results with variable phenotypic dominance and heterozygous hybrids. Here, eqs. 11 and 27 yield the prediction $V + V_\delta = (1 + 4v)p_{12}(1 - p_{12})$ where v is the variance associated with the dominance effects of fixed mutations (i.e., the δ_{ij} in eq. 25). Results differed systematically between simulations, because different evolutionary scenarios yield different values of v. All other details match Figure A2.5.

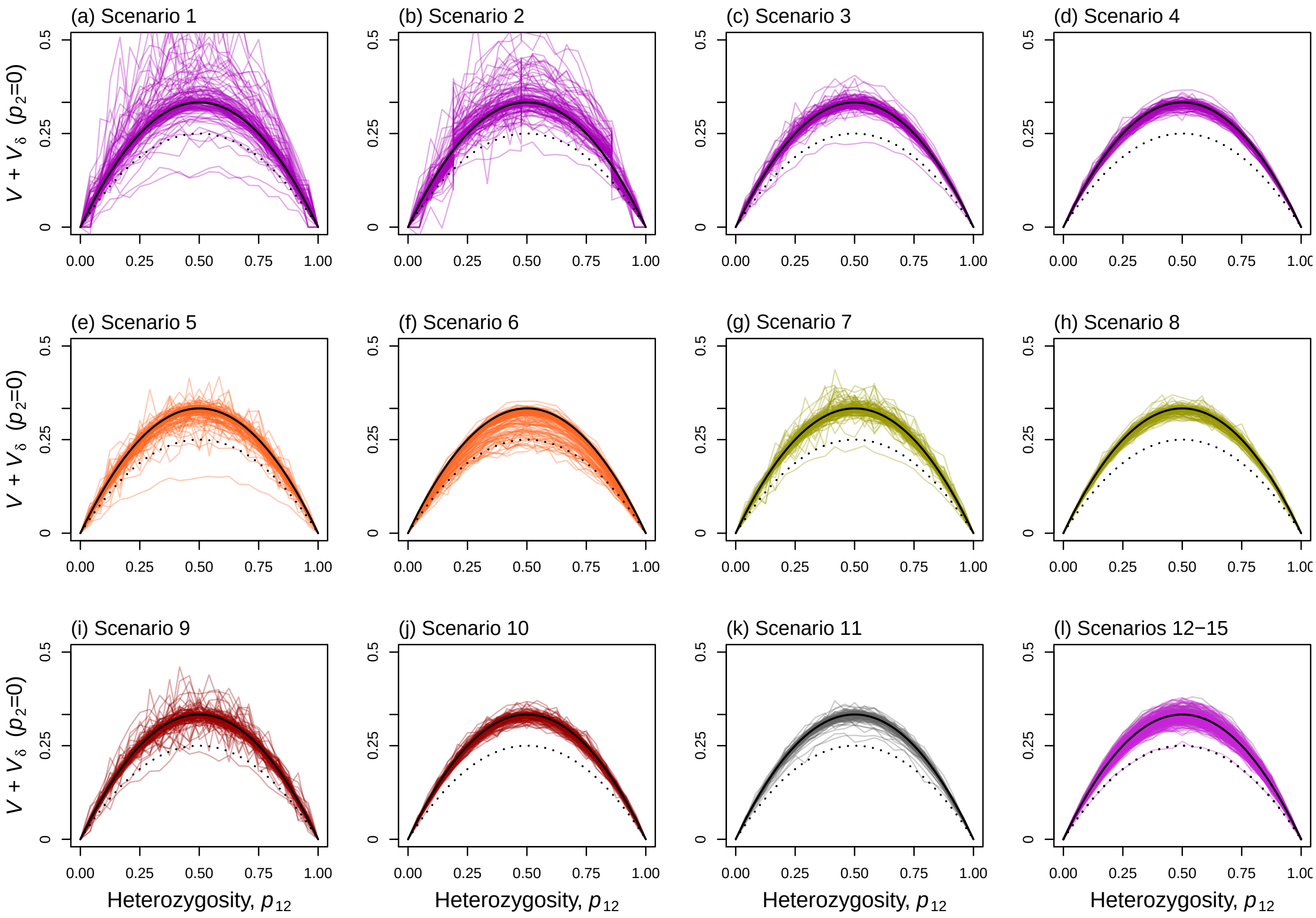

Figure A2.11 The Brownian bridge approximation for heterozygous hybrids, with variable phenotypic dominance. Plots replicate the results shown in Figure A2.10d, but separated according to the 15 divergence scenarios (see Figures A2.3 and A2.4 for details). Equations 11 and 27 yield the prediction $V + V_\delta = (1+4v)p_{12}(1-p_{12})$. Black solid lines show this prediction after setting $v = 1/12$, thereby matching the variance associated with the simulated mutation effects. Dotted lines show the predictions with $v = 0$, corresponding the additive phenotypic model.

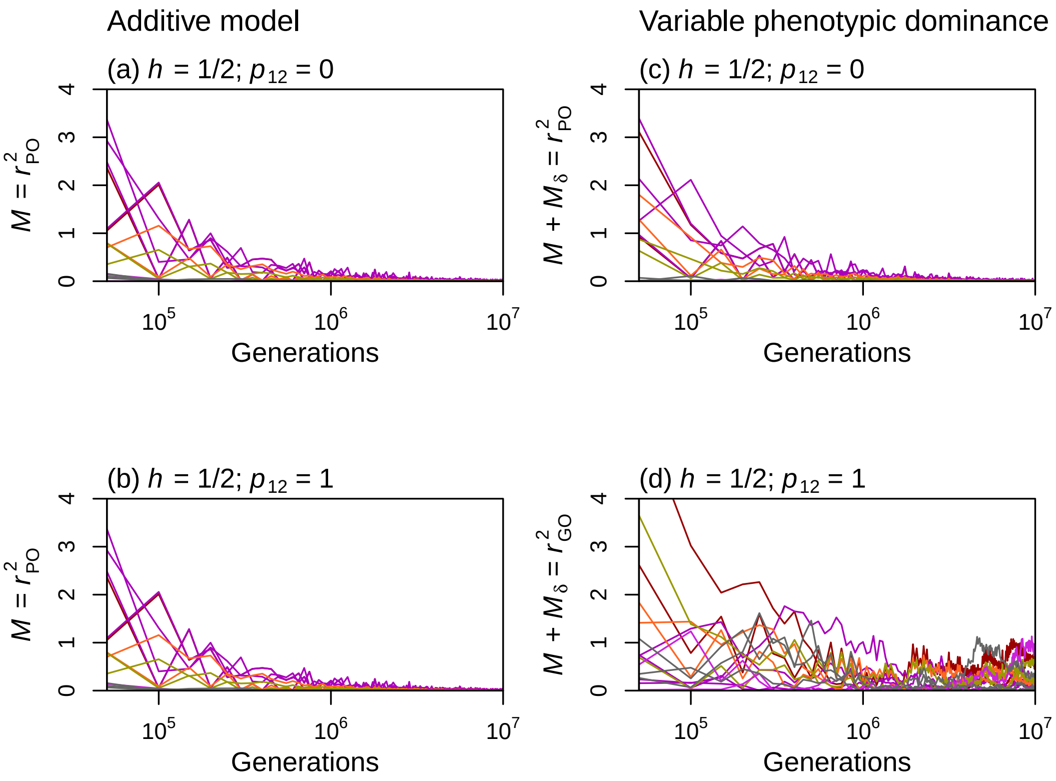

Figure A2.12 The second term of the Brownian bridge approximation, with and without phenotypic dominance. (**a**) For the additive model, M tends to decline over time, as long as genetic divergence increases. This causes convergence to the characteristic pattern of reproductive isolation (eq. 18). (**b**) For the additive model, M depends on h, but not on p_{12}, and so the same result applies to homozygous and heterozygous hybrids. (**c**) With variable phenotypic dominance, results for homozygous hybrids are unchanged. (**d**) But for heterozygous hybrids, the term never vanishes, even at very large levels of genomic divergence. All plots assume balanced hybrids ($h = 1/2$), so that the quantity plotted is equal to either r_{PO}^2 (the distance from the optimum of the midparental phenotype), or r_{GO}^2 (the distance from the optimum of the phenotype associated with the globally heterozygous genotype). $r_{\mathrm{PO}}^2 = r_{\mathrm{GO}}^2$ under additivity. Plots show results for all 15 divergence scenarios with an illustrative set of parameters: $k = 6$, $n = 2$, $NU = 1$, $Ns = -0.1$, bottom-up mutations, and free recombination.

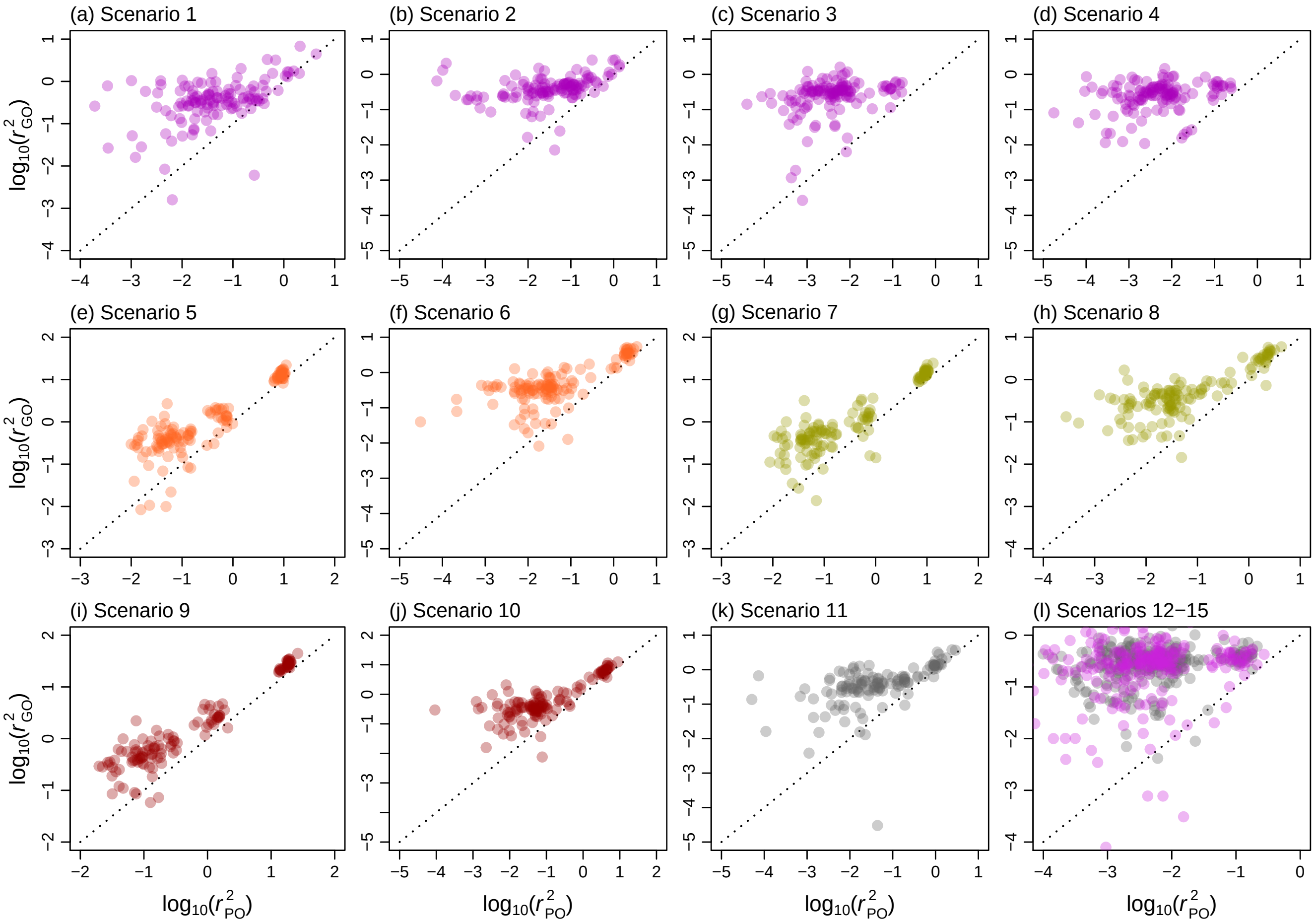

Figure A2.13 With variable phenotypic dominance, the phenotype of the global heterozygote, G, is usually further from the optimum than the midparental phenotype, P. This implies that the F1 may be unfit, even if the parental lines are optimally fit. Each plot shows simulation results under one or more of the 15 divergence scenarios (see Figures A2.3 and A2.4), with 128 different sets of population genetic parameters. There is variation in the results, but $r^2_{GO} > r^2_{PO}$ usually holds.

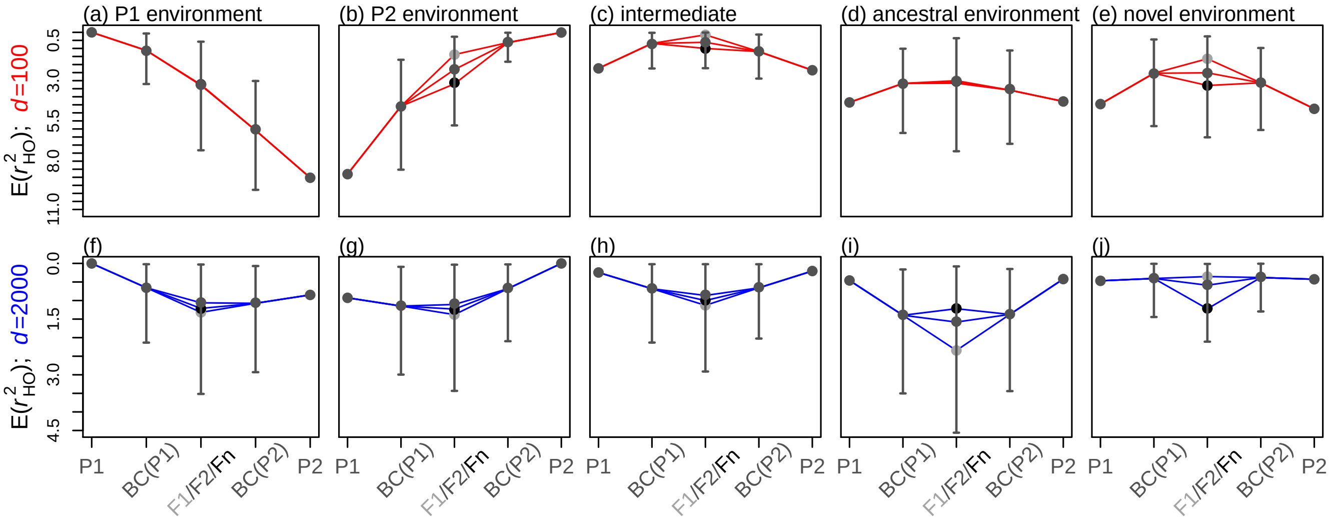

Figure A2.14 Simulated hybridization after local adaptation, with variable phenotypic dominance. All details match main text Figure 4, except that, during the process of divergence, simulated mutations had variable dominance effects. Compared to Figure 4, the main differences are (1) reduced fitness of the F1; (2) extrinsic (i.e., environment dependent) effects of heterozygosity and (3) directional dominance, such that reciprocal backcrosses have different fitness, even when the parental lines are equally fit. The red and blue lines used eqs. 26-28 with the simulated phenotypes of the parents and global heterozygote, and setting $v = 1/12$, to match the variance of the uniform distribution, which was used to generate the dominance effects of new mutations.
